# Modified Opposite-Spin-Scaled Double-Hybrid Functionals

**DOI:** 10.1021/acs.jpca.5c01035

**Published:** 2025-07-24

**Authors:** Golokesh Santra, Markus Bursch, Lukas Wittmann

**Affiliations:** † 28314 Max-Planck-Institut für Kohlenforschung, Kaiser-Wilhelm-Platz 1, 45470 Mülheim an der Ruhr, Germany; ‡ Interdisciplinary Center for Scientific Computing, Ruprecht-Karls-Universität Heidelberg, Im Neuenheimer Feld 205, 69120 Heidelberg, Germany; § FACCTs GmbH, 50677 Köln, Germany; ∥ Mulliken Center for Theoretical Chemistry, 9374 Universität Bonn, Beringstr. 4, 53115 Bonn, Germany

## Abstract

We investigate the potential performance improvements of double-hybrid density functionals by replacing the standard scaled opposite-spin MP2 (SOS-MP2) with the modified opposite-spin-scaled MP2 (MOS-MP2) in the nonlocal correlation component. Using the large and diverse GMTKN55 data set, we find that MOS-double hybrids provide significantly better accuracy compared to SOS-MP2-based double hybrids when empirical dispersion correction is not employed. The noncovalent interaction subsets account for the majority of this improvement. However, when the D4 dispersion correction is applied, the performance gap between MOS-MP2- and SOS-MP2-based double hybrids becomes negligible. While the new methods do not outperform the current state-of-the-art double hybrid functionals, our study offers valuable insights into the applicability of distance-dependent MP2 in place of conventional SOS-MP2, as well as the critical role of empirical dispersion corrections in further enhancing accuracy—insights that are useful for guiding future method developments. For nine transition metal sets, dispersion-corrected spin-component-scaled double hybrids are still significantly better than any MOS-double hybrid functional.

## Graphical abstract

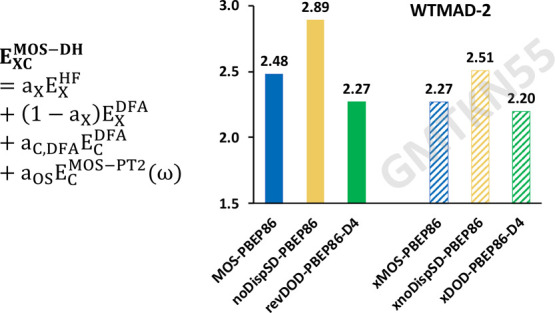

## 1 Introduction

Kohn–Sham density functional theory (DFT) is one of the main cornerstones of modern computational chemistry, valued for its ability to achieve a good balance between accuracy and computational efficiency, especially with increasing system size.^1,2^ Perdew’s *Jacob’s Ladder* categorizes density functionals based on the type of density or orbital information they utilize within the approximation.^3^ Each ascending rung on the ladder is believed to offer greater accuracy than the one before it, though this improvement typically comes with increased computational cost. The highest, fifth rung, is represented by the so-called double-hybrid (DH) functionals that include both, an admixture of Hartree–Fock exchange (HFx) and a wave function theory-based correlation contribution into the density functional formulation. The latter is usually computed by second-order perturbation theory (PT2), with second-order Mo̷ller-Plesset theory (MP2) being the most prominent method of choice.^4–8^

In his seminal paper, Grimme demonstrated that MP2 energies can be significantly improved by semiempirically scaling the opposite-spin (OS) and same-spin (SS) components using separate scaling factors.^9^ This method, termed spin-component-scaled MP2 (SCS-MP2), employed scaling parameters of 1.2 for the OS part and 0.33 for the SS part. Later, Head-Gordon and co-workers demonstrated that similar quality thermochemistry results can be obtained by only retaining and scaling the opposite spin correlation.^10^ Their method, known as scaled opposite-spin MP2 (SOS-MP2), is particularly interesting because the SOS-MP2 energy can be evaluated using the RI approximation combined with a Laplace transform technique, resulting in computational scaling of only the 4th power with respect to molecular size.

Motivated by these simple yet elegant approaches, such strategies have also been successfully incorporated into double-hybrid frameworks by various groups. When combined with empirical dispersion corrections, spin-scaled double hybrids often yield the best performance in benchmarks covering both main-group and transition-metal chemistry.^11–19^ Notable examples include the dispersion-corrected spin-component-scaled double hybrids (DSD) developed by Martin and co-workers;^14,15,20^ PWPB95 and more recently proposed r^2^SCAN-based double hybrids by the Grimme group;^21,22^ ωB97M(2) by Head-Gordon and co-workers^23^ ; nonempirical double hybrids by Adamo and co-workers;^24–26^ and the XYG-type double hybrids introduced by Xu and Goddard.^27^

Despite the popularity of MP2 as the nonlocal correlation component of DHs, it has inherent limitations, including its divergent behavior for small orbital energy gaps and underestimation of long-range dispersion interactions.

The first problem can be addressed by replacing simple MP2 with the direct random phase approximation^28^ (dRPA) correlation or by regularizing the MP2 energy expression, e.g., using the κ-regularization scheme proposed by Shee et al.^29^ In a recent study, we showed that dispersion-corrected dRPA-based double hybrids are only as good as MP2-based double hybrids for main group chemistry problems, while dRPA-based double hybrids perform significantly better for metal–organic barrier heights.^30^ The second possibility has also been investigated for the double-hybrid functionals.^22,31^ Martin and co-workers have shown that for typical double hybrids, which usually employ a large percentage (∼70% or higher) of HF-exchange, using κ-MP2 correlation instead of canonical MP2 has no extra benefit.^31^ The regularized DHs are better than their standard counterparts, only if a smaller percentage of HF-exchange (∼50%) is used without the spin-component scaling of the MP2 energy. In a more recent study, Wittmann et al. have found that κPr^2^SCAN50, employing only 50% HFx, performs marginally better overall than the regular Pr^2^SCAN50, while improving performance for small orbital-energy gap systems and thus adding a layer of robustness for such systems.^22^

The second problem is often addressed by adding a dispersion correction to the total electronic energy. This is a common issue with many density functional approximations (DFAs), as they often fail to accurately describe long-range correlation effects, leading to a systematic underestimation of London dispersion interactions. As double hybrids usually contain a scaled-down portion of MP2 correlation, they also suffer from a similar issue; therefore, additional London dispersion corrections are required. Some popular dispersion correction schemes include Grimme’s D3 ^32,33^ and D4 ^34–37^ corrections, different variants of Vydrov and van Voorhis’s VV10 model,^38–40^ the exchange-hole dipole moment (XDM) model of Becke and Johnson,^41–44^ or the Tkatchenko–Scheffler (TS) method.^45,46^ Specifically, the efficient DFT-D approach has proven reliable in countless quantum chemical applications and workflows.^47–50^

Even though both spin-component-scaled (SCS) MP2 and scaled opposite spin (SOS) MP2 provide satisfactory results within short and medium ranges, they fail to correctly describe the long-range correlation. To address this issue, Head-Gordon and co-workers proposed a distance-dependent modification, where they split the electron interaction operator (1/*r*_12_) of SOS-MP2 into a short- and a long-range part.^51^ In the short-range regime, the MP2 energy is scaled by the usual factor of 1.3, but the long-range scaling factor is adjusted to 2.0 – which recovers the asymptotic MP2 interaction energy of distant fragments. This modified opposite-spin MP2 (MOS-MP2) has been shown to provide a significant improvement over SOS-MP2 for a variety of chemical problems involving both short-range and long-range interactions.^51^

The primary objective of this study is to demonstrate that extending the double-hybrid framework to incorporate MOS-MP2 is feasible and enhances performance compared to SOS-MP2-based counterparts, potentially inspiring further advancements in the field. Moreover, we shall investigate whether the modified opposite-spin-scaled double hybrids outperform their corresponding SOS-MP2-based counterparts when empirical dispersion correction is added.

## 2 Theory

### 2.1 MOS-MP2

Due to the use of a fixed scaling factor, scaled opposite spin MP2 is known to systematically underestimate long-range correlation. To address this limitation, Head-Gordon and co-workers proposed a modified SOS-MP2 scheme where the scaling factor is distance-dependent.^51^ This adjustment introduces a range-separation scheme similar to that used in Hartree–Fock exchange but applied to the MP2 correlation. The exchange operator in the MP2 integrals is replaced by the MOS operator

$${\hat{g}}_{\omega }(r)=\frac{1}{r}+c_{\mathrm{MOS}}\frac{\mathrm{erf}(\omega r)}{r}$$ 1

where ω determines the strength of attenuation. It leads to the modified integral expression:

$${\tilde{I}}_{ia}^{jb}=\int dr\int dr^{\prime }\phi_{i}(r)\phi_{a}(r){\hat{g}}_{\omega }(r-r^{\prime })\phi_{j}(r^{\prime })\phi_{b}(r^{\prime })$$ 2

which results the following expression for MOS-MP2 correlation energy

$$E_{\mathrm{MP}2}^{\mathrm{MOS}}=-\sum_{ia}^{\alpha }\sum_{jb}^{\beta }\frac{{\tilde{I}}_{ia}^{jb}(\omega ){\tilde{I}}_{ia}^{jb}(\omega )}{\left[\epsilon_{a}+\epsilon_{b}-\epsilon_{i}-\epsilon_{j}\right]}$$ 3

where *i*, *j* are the occupied and *a*, *b* are the virtual orbitals with corresponding eigenvalues, ϵ. The MOS operator, *ĝ*_ω_(**r**), (eq 1) depends on two parameters, ω and *c*_MOS_. *c*_MOS_ can be fixed at $\sqrt{2}-1$ by using the condition of

$$\lim_{r\to \infty }\;E_{\mathrm{MP}2}^{\mathrm{MOS}}=2E_{\mathrm{MP}2}^{\mathrm{OS}}$$ 4

Hence, our MOS-MP2 scheme depends only on a single parameter, ω. For main group chemistry problems, the Lochan, Jung, and Head-Gordon recommends ω = 0.6. Similar to SOS-MP2,^10^ for MOS-MP2, the scaling factor for the same-spin MP2 correlation energy components is 0, but the parameter for the opposite-spin MP2 correlation ranges from 1.3 to 2.0.

### 2.2 Double Hybrids: Hartree–Fock and MP2 Admixture

In 2006, Grimme proposed the so-called *double hybrid* functionals by combining a fraction of exact exchange and nonlocal GLPT2 ^52^ (second-order Görling–Levy perturbation theory) correlation with the semilocal DFT exchange and correlation components.^4^ These functionals have the following expression for the exchange-correlation energy

$$E_{\mathrm{XC}}^{\mathrm{DH}}=a_{X}E_{X}^{\mathrm{HF}}+(1-a_{X})E_{X}^{\mathrm{DFA}}+(1-a_{C})E_{C}^{\mathrm{DFA}}+a_{C}E_{C}^{\mathrm{MP}2}$$ 5

where *E*_X_^DFA^ and *E*_C_^DFA^ represent the semilocal exchange and correlation energy components; *E*_X_^HF^ and *E*_C_^MP2^ are the HF-exchange and GLPT2 correlation energies – *a*_X_ and *a*_C_ are the respective parameters. Previously, the term gDH was used for these types of functionals.^53^ Later, Martin and co-workers showed that using separate parameters for the same and opposite-spin MP2 correlation (i.e., *a*_OS_ and *a*_SS_) improved the accuracy of DHs for main-group thermochemistry and harmonic frequencies.^14,15,20,54–56^

Another family of DHs, often referred to as xDHs, uses full semilocal correlation instead to generate KS reference orbitals.^53,57,58^ It was argued that such orbitals are more appropriate as a basis for GLPT2 than the damped-correlation orbitals in the gDHs.^7,59^ However, this argument has been refuted on empirical grounds by Goerigk and Grimme.^11^ The XYG-family of double hybrids from Xu and Goddard, and the xDSD and xDOD functionals by Martin and co-workers belong to that category.^7,27,53,57–62^

The exchange-correlation energy for a modified opposite-spin scaled double hybrid (MOS-DH) functional is expressed as

$$E_{\mathrm{XC}}^{\mathrm{MOS}‐\mathrm{DH}}=a_{X}E_{X}^{\mathrm{HF}}+(1-a_{X})E_{X}^{\mathrm{DFA}}+a_{C,\mathrm{DFA}}E_{C}^{\mathrm{DFA}}+a_{\mathrm{OS}}E_{C}^{\mathrm{MOS}‐\mathrm{MP}2}$$ 6

where *E*_X_^DFA^, *E*_X_^HF^, and *E*_C_^DFA^ represent the same energy component as in eq 5. *a*_X_ and *a*_C,DFA_ are the parameters for the HF-exchange and the semilocal-correlation energy components. *E*_C_^MOS‑MP2^ is the MOS-MP2 correlation energy component, and *a*_OS_ is the corresponding parameter. We refer to these new functionals as MOS_n_-XC in the remaining text, where XC is a combination of DFA exchange and correlation and *n* is the percentage of HF-exchange (i.e., *n* = 100*a*_X_). In passing, we must note that *a*_C,DFA_ in eq 6 and (1 – *a*_C_) in eq 5 are the same parameters.

### 2.3 DFT-D4 Dispersion Correction

The default atomic-charge dependent D4 dispersion correction including Axilrod–Teller–Muto^63,64^ (ATM) type three-body contributions was applied according to eqs 7a and 7b with atomic indices *A*, *B*, and *C*, their distance *R*_*AB*_, the *n*th dispersion coefficient *C*_(*n*)_^*AB*^, and the angle-dependent term θ_ABC_

$$E_{\mathrm{disp}}^{D4}=-\frac{1}{2}\sum_{AB}\sum_{n=6,8}s_{n}\frac{C_{\left(n\right)}^{AB}}{R_{AB}^{\left(n\right)}}f_{\mathrm{damp}}^{\left(n\right)}(R_{AB})$$ 7a

$$-\frac{1}{6}\sum_{ABC}s_{9}\frac{C_{\left(9\right)}^{ABC}}{R_{ABC}^{\left(9\right)}}f_{\mathrm{damp}}^{\left(9\right)}(R_{ABC},\theta_{ABC})$$ 7b

where *f*_BJ_^(*n*)^(*R*_*AB*_) corresponds to the default Becke–Johnson (BJ) damping function^33^ according to eq 8:

$$f_{\mathrm{BJ}}^{\left(n\right)}(R_{AB})=\frac{R_{AB}^{\left(n\right)}}{R_{AB}^{\left(n\right)}+(a_{1}R_{0}^{AB}+a_{2}{)}^{\left(n\right)}}$$ 8

The usually fitted parameters for a non-DH functional are *s*_8_, *a*_1_, and *a*_2_. For a DH, *s*_6_ must also be adjusted due to the presence of the MP2 correlation term.

Additionally, *s*_9_ is also an adjustable parameter, but its optimization requires a data set containing larger structures where the Axilrod–Teller–Muto (ATM) contribution is significant – something that is absent in GMTKN55. Typically, *s*_9_ is set to unity to guarantee the reasonable behavior of the dispersion correction in the asymptotic limit.^34,37,65,66^ Following refs 53 and 22, we impose the constraint *s*_9_ = 1 while optimizing the parameters for dispersion-corrected MOS-MP2-based double hybrids. The topic is, however, not straightforward, and more can be found in, e.g., refs 67–70 .

## 3 Computational Details

Unless otherwise specified, all calculations were performed using the Q-Chem 5.4 quantum chemistry program package.^71^ The Weigend-Ahlrichs quadruple-ζ basis set def2-QZVPP^72^ was used for all calculations. For seven GMTKN55 subsets (WATER27, RG18, IL16, G21EA, AHB21, BH76, and BH76RC) the diffuse-function-augmented def2-QZVPPD^73^ was employed instead. The matching effective core potentials (ECPs)^74,75^ for heavy elements with *Z* > 36 were generally employed. For the MP2 part, RI approximation was applied to accelerate the calculations in conjunction with the def2-QZVPPD-RI^76,77^ auxiliary basis set. The SG-3 ^78^ integration grid was employed, except for the SCAN (strongly constrained and appropriately normed) variants,^79^ where an unpruned (150, 590) grid was used for its severe integration grid sensitivity.^80^

DFT-D4 dispersion corrections were calculated with the dftd4 3.4.0 standalone program.^34–37^

Reference geometries for the GMTKN55 benchmark sets were taken from ref 12. All 55 subsets present in GMTKN55 are explained with appropriate references in Table S1 of Supporting Information.

Additionally, nine transition-metal chemistry sets CUAGAU-2 (atomization, ionization, isomerization, and binding energies for copper, silver, and gold clusters),^81^ LTMBH (activation energies for the late-transition-metal-catalyzed reactions),^82^ MOBH35 (forward and reverse metal–organic barrier heights, 35 reactions),^17,83^ MOR41 (reaction energies of 41 closed-shell organometallic reactions),^19^ ROST61 (reaction energies of 61 open-shell single-reference transition metal complexes),^18^ TMBH (activation energies of Zr, Mo, Ru, Rh, W, and Re catalyzed organic reactions),^84–87^ TMCONF16 (conformational energies of transition metal complexes),^88^ TMIP (ionization energies of first-row transition metal complexes in the +2 or +3 oxidation state, with either cyclopentadienyl or acetylacetonate ligands),^89^ and WCCR10 (ligand dissociation energies of large transition-metal complexes)^90,91^ were also evaluated. This compilation of benchmark sets will be referred to as “TM9” throughout this paper.

## 4 Parametrization Strategy

The modified opposite-spin scaled double hybrid functionals have been parametrized using the GMTKN55 benchmark suite.^12^ This data set consists of 55 types of chemical problems, which can be further divided into five subsets: basic thermochemistry of small molecules, barrier heights, large molecule reactions, intermolecular interactions, and conformer energies.

Originally proposed by Goerigk et al., the WTMAD-2 ^12^ (weighted total mean absolute deviation) has been used as the primary metric for the performance evaluation and parameter optimization of the MOS-DHs. From a statistical viewpoint, MAD (mean absolute deviation) is a more robust metric than RMSD (root-mean-square deviation), as the former is more resilient to a small number of large outliers than the latter.^92^ For a normal distribution without a systematic error, RMSD $\approx \frac{5}{4}\mathrm{MAD}$ .^93^ See Appendix B in the Supporting Information for the definition of the used statistical measures.

The constructed MOS-DH functionals have four empirical parameters: (i) fraction of exact exchange (*a*_X_); (ii) fraction of the semilocal DFT correlation (*a*_C,DFA_); (iii) coefficient for the MOS-MP2 correlation (*a*_OS_); and (iv) the parameter ω, which regulates the attenuation level of the MOS operator.

Powell’s BOBYQA (bound optimization by quadratic approximation) derivative-free constrained optimizer was used for the optimization.^94^ For a given set of {*a*_X_, *a*_C,DFA_, ω}, it is possible to obtain the optimal value of *a*_OS_ without any further electronic structure calculations simply by extracting individual energy components from the calculations, evaluating total energies and hence WTMAD-2 for a given *a*_OS_, and minimizing WTMAD-2 with respect to *a*_OS_ using BOBYQA. It can be considered as the *microiteration* loop. In comparison, the outer *macroiteration* loop consists of varying {*a*_X_, *a*_C,DFA_, ω} and reevaluating the full GMTKN55 using the updated set of parameters. For the revised DSD and DOD functionals, we found that *a*_C,DFA_ could be safely included in the microiteration, but *a*_X_ could not due to its strong coupling with the MP2 scaling factors.^14^ Hence, we have adopted the practice of microiterating {*a*_C,DFA_, *a*_OS_} at every macroiteration using BOBYQA. We must note that, with full microiteration cycles, additional macroiterations beyond the first typically do not have significantly improved performance unless the starting guess is especially poor. Hence, the output of the first cycle is reported. The optimum value of ω for each *a*_X_ was determined manually by interpolation.

For each exchange-correlation combination, the above-mentioned process is repeated with multiple *a*_X_ values to find the best MOS-DH, which offers the lowest WTMAD-2.

While using D4 dispersion correction with MOS-DHs, four extra parameters needed to be included in the microiteration loop: *s*_6_, *s*_8_, *s*_9_, *a*_1_, and *a*_2_. Hence, for each {*a*_X_, ω} set, we optimized {*a*_C,DFA_, *a*_OS_, *s*_6_, *s*_8_, *s*_9_, *a*_1_, *a*_2_}. Like in revDOD-PBEP86-D4 and xDOD_75_-PBEP86-D4, the *s*_9_ = 1 constraint was used across the board while optimizing the other microiteration parameters of MOS-DH-D4 functionals.

## 5 Results and Discussion

### 5.1 Main Group Chemistry Problems (GMTKN55)

To assess the impact of using MOS-MP2 as the nonlocal correlation component in double hybrids, we replaced the SOS-MP2 parts of noDispOD_69_-PBEP86 and xnoDispOD_72_-PBEP86 with MOS-MP2 and reoptimized the corresponding parameters. As a result, the WTMAD-2_GMTKN55_ values improve by 1.25 and 1.17 kcal mol^–1^, respectively (Table S2 in the Supporting Information). The lion’s share of this improvement in both cases originates from basic thermochemistry and noncovalent interactions (see Figure 1). Further analysis of all 55 subsets in GMTKN55 reveals that the W4–11, PCONF21, and S66 subsets show significant improvements (Table S3 in the Supporting Information). As MOS-MP2 uses a distance-dependent scaling factor (ranging from 1.3 to 2.0), the optimized *a*_OS_ values for the MOS-MP2-based double hybrids are consistently smaller than that of their SOS-MP2 counterparts. For instance, when comparing noDispOD_69_-PBEP86 to its MOS-MP2-based counterpart, the ratio of their *a*_OS_ parameters is 1.4, which rises to 1.7 as *a*_X_ increases from 0.69 to 0.82. To investigate how the distance-dependent scaling factor (ω) in MOS-MP2 affects the performance of double hybrids, we examined two dispersion-dominated subsets, RG18 and ADIM6. In both cases, varying ω significantly influenced the accuracy of MOS69-PBEP86. Compared to its SOS-MP2-based counterpart, noDispOD69-PBEP86, MOS_69_PBEP86 achieved the best accuracy with ω = 0.4 (see Figure S1 in the Supporting Information).

**Figure 1:**
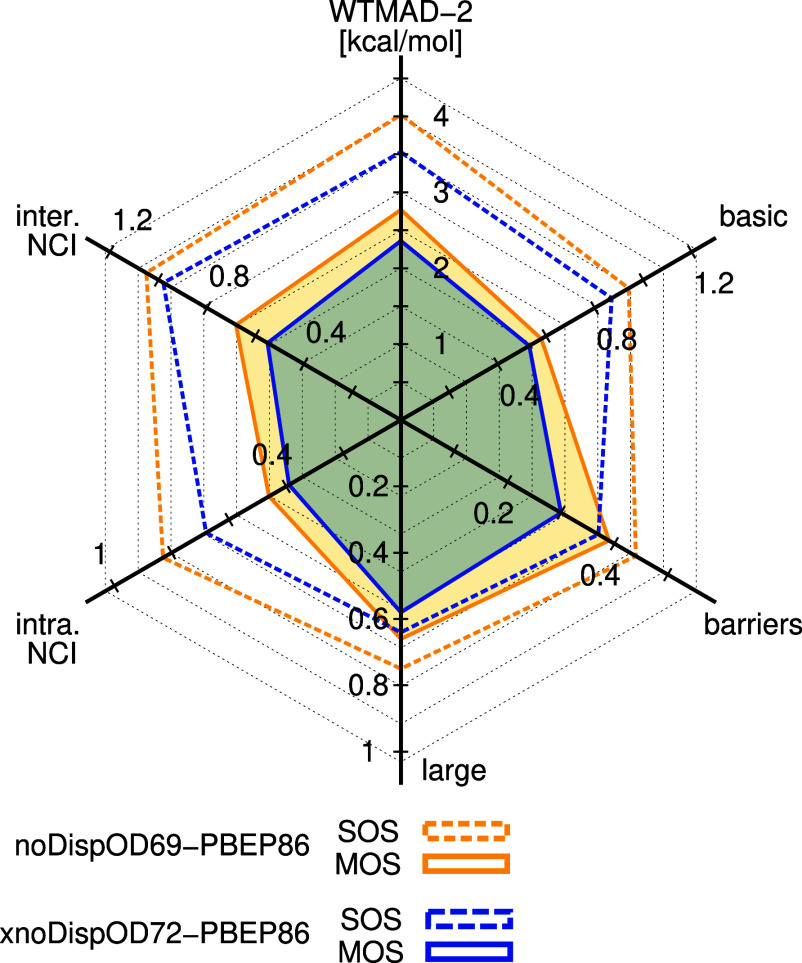
Effect of using MOS-MP2 in a double hybrid functional on WTMAD-2_GMTKN55_ and contributions from five major subsets of GMTKN55 (i.e., ΔWTMAD-2).

Using MOS-MP2 instead of SOS-MP2 noticeably enhanced the accuracy of dispersion-uncorrected double hybrids. This prompted us to further optimize all parameters of the MOS-DH functionals. The final parameters and WTMAD-2_GMTKN55_ values for the GMTKN55 for various MOS-DHs and their corresponding SCS-MP2-based dispersion-free counterparts are listed in Table 1.

**Table 1:** Final Parameters and Total WTMAD-2_GMTKN55_ (in kcal mol^–1^) of the MOS-DHs and Their Respective Spin-Component-Scaled, Dispersion Uncorrected DHs on the GMTKN55

**functional**	**WTMAD-2**_**GMTKN55**_	ω	*a*_*X*_	*a*_*X*,DFA_	*a*_*C*,DFA_	*a*_*OS*_	*a*_*SS*_
MOS_76_-PBEP86	2.48	0.50	0.76	0.24	0.4371	0.5602	[0]
xMOS_78_-PBEP86	2.27	0.65	0.78	0.22	0.4056	0.5373	[0]
noDispSD_82_-PBEP86	2.89		0.82	0.18	0.3073	0.7426	0.3782
xnoDispSD_82_-PBEP86	2.51		0.82	0.18	0.2797	0.7678	0.3521

While using the PBE exchange and P86 correlation, varying the percentage of HF-exchange and the MOS-MP2 rang-separation parameter (ω) simultaneously, we got the lowest WTMAD-2_GMTKN55_ of 2.48 kcal mol^–1^ with *a*_X_ = 0.76 and ω = 0.50 (see Figure 2 and Table 1). Among the five major subcategories of the GMTKN55 benchmark, barrier heights and noncovalent interactions benefit the most upon using MOS-MP2 (see Table 2). When compared to the revDOD-PBEP86-D4, the absence of empirical dispersion correction significantly deteriorates the accuracy of MOS_76_-PBEP86 for the pericyclic reaction barrier heights, BH76, RSE43, and RG18 subsets, but outperforms revDOD-PBEP86-D4 for the tautomer relative energies (Table S4 in the Supporting Information).

**Table 2:** Total WTMAD-2_GMTKN55_ and the Contributions from the Five Major Subsets, Denoted as ΔWTMAD-2

		Δ**WTMAD-2** (**kcal** **mol** ^–1^)				
**functional**	**total WTMAD2** (**kcal** **mol** ^–1^)	**basic thermochemistry**	**barrier heights**	**large molecule reactions**	**intramolecular NCI** ^a^	**intermolecular NCI** ^a^
MOS_76_-PBEP86	2.48	0.52	0.37	0.64	0.41	0.55
xMOS_78_-PBEP86	2.27	0.52	0.32	0.54	0.38	0.51
noDispSD_82_-PBEP86	2.89	0.57	0.49	0.65	0.51	0.66
xnoDispSD_82_-PBEP86	2.51	0.52	0.40	0.53	0.46	0.61
revDOD-PBEP86-D4 ^53^	2.27	0.58	0.25	0.58	0.40	0.46
xDOD_72_-PBEP86-D4 ^53,62^	2.20	0.57	0.23	0.51	0.41	0.47
Pr^2^SCAN69-D4 ^22^	2.72^b^	0.62	0.36	0.58	0.59	0.56

a NCI = noncovalent interactions.

b Due to the use of def2-QZVPPD basis set for seven GMTKN55 subsets (WATER27, RG18, IL16, G21EA, AHB21, BH76, and BH76RC) in the present work, the total WTMAD-2_GMTKN55_ is 0.09 kcal/mol lower than what is reported in ref 22.

**Figure 2:**
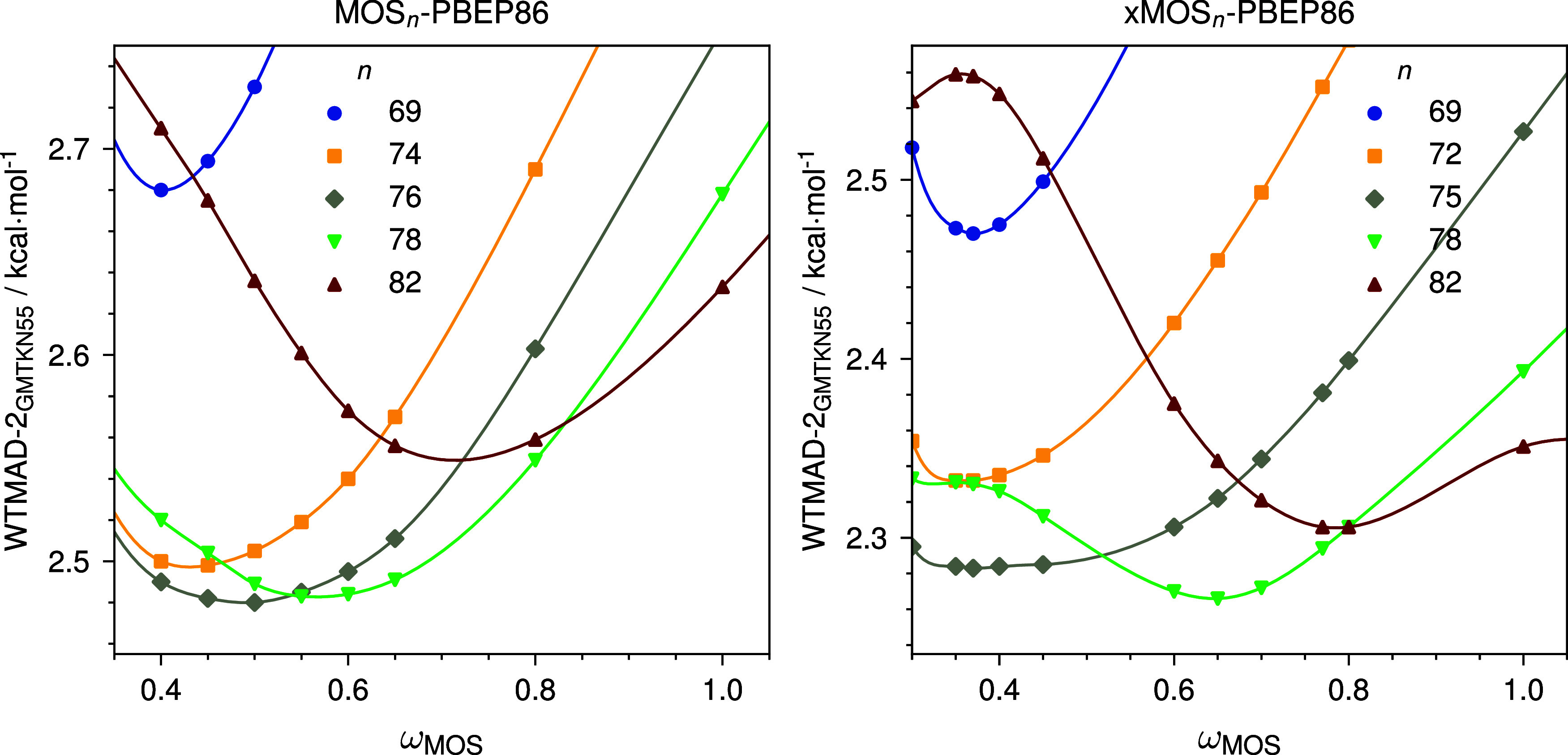
Dependence of WTMAD-2_GMTKN55_(kcal mol^–1^) on the MOS-MP2 range-separation parameter ω for MOS_n_-PBEP86 and xMOS_n_-PBEP86.

The interaction energies of rare gas dimers and trimers are primarily governed by dispersion forces. In an analysis using six such reactions from the RG18 set, the revDOD-PBEP86-D4 functional demonstrates a mean absolute deviation (MAD) of only 0.08 kcal mol^–1^ compared to the CCSD­(T) reference. However, noDispSD_82_-PBEP86 exhibits a significantly higher MAD (0.16 kcal mol^–1^). Meanwhile, MOS_76_-PBEP86 exhibits intermediate accuracy, with a mean error of 0.13 kcal mol^–1^. In passing, we note that all three methods systematically underestimate those interaction energies (see Figure S2 in the Supporting Information).

For a specific MOS-MP2 range-separation parameter (ω), an increasing percentage of HF-exchange yields a larger optimized value of the MOS-MP2 correlation parameter *a*_OS_, while the fraction of the semilocal DFT correlation decreases (Figure 3). On the other hand, for a fixed value of *a*_X_, increasing ω yields a lower *a*_OS_. For a very small ω (i.e., ω < 0.5) *a*_C,DFA_ decreases (Table S11 in the Supporting Information). The trend of the WTMAD-2_GMTKN55_ against the optimized *a*_OS_ is shown in Figure S3 in the Supporting Information.

**Figure 3:**
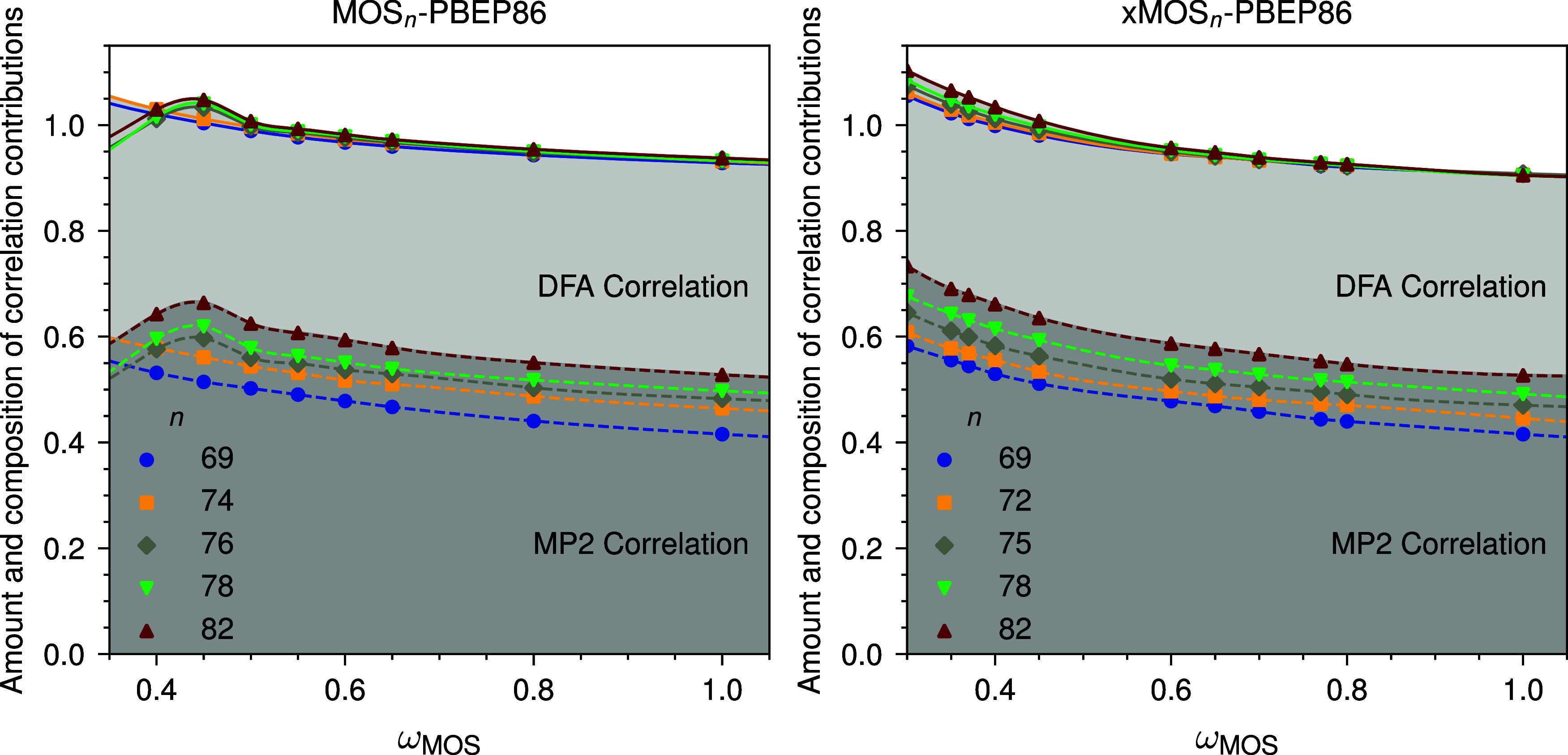
Dependence of the optimized amounts of MOS-MP2 and DFA correlation on the MOS-MP2 range-separation parameter ω for MOS_n_-PBEP86 and xMOS_n_-PBEP86.

The exchange-correlation energy expression of MOS-DHs might suggest that adding the a_OS_ scaling prefactor in eq 6 disrupts the correct asymptotic dispersion limit that MOS-MP2 follows. However, by plotting the interaction energy of stretched Ar_2_, we found that the proper asymptotic *R* ^–6^ dependence is still maintained in MOS_76_-PBEP86 (see Figure S4 in the Supporting Information). Further evaluation on the S66 × 8 benchmark suggests that the performance of MOS- and xMOS-DHs lies between that of dispersion-uncorrected and dispersion-corrected SCS-MP2-based double hybrids (Table S25 in the Supporting Information). The errors are largest for compressed geometries and decrease with the stretching of intermolecular distances. At 2.0*r*_e_, there is very little difference between the performance of the tested functionals.

Among the several exchange-correlation combinations tested for the revDSD- and revDOD-family double hybrids, the lowest WTMAD-2 for the GMTKN55 benchmark was achieved with PBE-P86.^14^ Similarly, for the MOS-DHs, the SCAN–SCAN and PBE–PBE combinations exhibited poorer performance compared to their PBE-P86 counterparts. For the MOS_n_-PBE functionals, the lowest WTMAD-2 error was obtained with *a*_X_ = 0.78 and ω = 0.50, while for the MOS_n_-SCAN functionals, the optimal values of these parameters were *a*_X_ = 0.74 and ω = 0.90 (see Table S5 and Figure S5 in the Supporting Information). For each {*a*_X_, ω} combination, the WTMAD-2_GMTKN55_ error statistics for the SCAN- and PBE-based MOS-DHs are provided in Tables S9 and S10 in the Supporting Information, respectively.

For the xDH variants of MOS_n_-PBEP86, we obtained the lowest WTMAD-2_GMTKN55_ of 2.27 kcal mol^–1^ by employing *a*_X_ = 0.78 and ω = 0.65 (Figure 2). Transitioning from the gDH to the xDH variant results in a WTMAD-2 improvement of 0.21 kcal mol^–1^, for the MOS-MP2-based double hybrids, but for the noDispSD functionals, that improvement is 0.37 kcal mol^–1^ (Table 1). The majority of that improvement for MOS-DHs originates from the large-species reaction energies. A detailed analysis of the 55 subsets reveals that, for the RSE43 set, xMOS78-PBEP86 performs noticeably better than MOS76-PBEP86. For a fixed ω, increasing the amount of HF exchange correlates with higher *a*_OS_ and correspondingly lower *a*_C,DFA_ values (see Figure 3 and Table S10 in the Supporting Information). A comparison of the WTMAD-2 errors relative to the optimized *a*_OS_ values is provided in Figure S2 of the Supporting Information.

Across five major subsets of GMTKN55, xMOS_78_-PBEP86 demonstrated significant advantages over xnoDispSD_82_-PBEP86 for barrier heights and noncovalent interactions. However, xnoDispSD_82_-PBEP86 significantly outperforms its competitor for the reactions in W4–11, MB16–43, and BSR36 (Table S4 in the Supporting Information).

Interestingly enough, the WTMAD-2_GMTKN55_ gap between xMOS_78_-PBEP86 and xDOD_72_-PBEP86-D4 is only 0.07 kcal mol^–1^. Except for the basic thermochemistry and intramolecular noncovalent interactions, xDOD_72_-PBEP86-D4 outperforms xMOS_78_-PBEP86 for the remaining reaction categories of GMTKN55 (Tables 2 and S6 in the Supporting Information).

For a fixed value of *a*_X_, the remaining parameters of xMOS_n_-PBEP86 and xDOD_n_-PBEP86-D4 are optimized and their performance evaluated. The WTMAD-2_GMTKN55_ gap decreases progressively as *a*_X_ increases from 0.50 to 0.78. Beyond 78% HF-exchange, MOS-DHs outperform the corresponding xDOD-D4 functionals (see Figure S6 and Table S15 in the Supporting Information). Only for the basic thermochemistry reactions, xMOS-DHs show superior performance compared to xDOD-D4, with the performance gap widening as %HF exchange increases. For the remaining four reaction types of GMTKN55, xDOD-D4 functionals surpass xMOS-DHs at a smaller percentage of HF exchange. However, at a larger percentage (e.g., *a*_X_ = 0.85), xMOS-DH and xDOD-D4 offer similar accuracy (Figure S6 in the Supporting Information). The optimized parameters for xMOS_n_-PBEP86 and xDOD_n_-PBEP86-D4 are listed in Tables S14 and S14 in the Supporting Information.

An increasing proportion of HF admixture results in a smaller performance difference between xMOS_n_-PBEP86 and xnoDispOD_n_-PBEP86. For basic thermochemical, the WTMAD-2 difference between xMOS_50_-PBEP86 and xnoDispOD_50_-PBEP86 is minimal, but it grows as the percentage of Hartree–Fock exchange rises. In contrast, for the other four subsets, the error gap gradually decreases as the amount of *a*_X_ increases (see Figure S6 in the Supporting Information).

For all MOS-DH functionals, the sum of the coefficients *a*_C,DFA_ and *a*_OS_ is nearly equal to one. Imposing the constraint *a*_C,DFA_ + *a*_OS_ = 1.0 during optimization reduces the number of empirically fitted parameters from four to three, but it does not significantly impact the performance of these methods. Refer to Table S19 in the Supporting Information for the final parameters and WTMAD-2_GMTKN55_ errors. The only exception is MOS_78_-PBE, which shows an increase in WTMAD-2 of 0.15 kcal mol^–1^. Most of this performance decline can be attributed to the S66 noncovalent interaction set.

When dispersion correction is not considered, the MOS-DHs are significantly better performers compared to Grimme’s B2PLYP functional. On the other hand, Zhang and Xu’s XYG7 has significantly lower WTMAD-2 than the new MOS-DHs.

### 5.2 Effect of Including D4 Dispersion Correction

For this purpose, we use MOS_n_-PBEP86 and xMOS_n_-PBEP86 series, combined with the DFT-D4 dispersion correction.

Contrary to refs 14 and 53, the optimized *s*_8_ parameters of MOS_n_-PBEP86 do not consistently approach zero. For a specific percentage of HF exchange, that parameter only vanishes for small ω values. Adding a dispersion correction to MOS_76_-PBEP86 (ω = 0.5) and reoptimizing the parameters leads to an overall improvement of 0.1 kcal mol^–1^ in total WTMAD-2 (see Table 3). A detailed analysis of all 55 subsets in GMTKN55 reveals that a major chunk of this improvement is driven by the RG18, ADIM6, MCONF, and PCONF21 subsets. In contrast, the inclusion of dispersion correction slightly worsens the performance for the W4–11 subset (Table S18 in the Supporting Information). Moreover, by simultaneously tuning the MOS-MP2 attenuation parameter (ω) and the fraction of exact exchange (*a*_X_), achieved the lowest WTMAD-2 value of 2.27 kcal mol^–1^ for MOS_69_-PBEP86-D4 (ω = 0.1). This value is identical to the WTMAD-2 obtained for revDOD-PBEP86-D4 (see Table 3).

**Table 3:** Total WTMAD-2 (in kcal mol^–1^) and Optimized Parameters for Dispersion-Uncorrected and Corrected MOS-DHs^a^,^b^

functionals	WTMAD-2	*a*_X_	ω	*a*_C,DFA_	*a*_OS_	s_6_	*s*_8_	*s*_9_	*a*_1_	*a*_2_
MOS_76_-PBEP86	2.48	0.76	0.50	0.4371	0.5602					
MOS_69_-PBEP86-D4	2.27	0.69	0.10	0.4340	0.6063	0.6134	–0.0377	[1.0]	0.3404	4.2066
MOS_76_-PBEP86-D4^c^	2.38	0.76	0.50	0.4188	0.5548	0.4034	–0.3954	[1.0]	0.6759	2.5184
revDOD-PBEP86-D4 ^14,53^	2.27	0.69		0.4301	0.6131	0.6158	[0]	[1.0]	0.3440	4.2426
xMOS_78_-PBEP86	2.27	0.78	0.65	0.4056	0.5373					
xMOS_78_-PBEP86-D4	2.19	0.78	0.80	0.3990	0.4965	0.1672	0.0023	[1.0]	0.3276	4.8708
xMOS_78_-PBEP86-D4^d^	2.21	0.78	0.65	0.3962	0.5368	0.1597	–0.1061	[1.0]	0.4611	4.0050
xDOD_72_-PBEP86-D4 ^14^	2.20	0.72		0.3999	0.6743	0.5395	[0]	[1.0]	0.2095	5.0154

a The parameters in the square bracket are kept constant while optimizing parameters.

b For comparison, corresponding dispersion-corrected SOS-MP2-based double hybrids are also included.

c The D4 dispersion corrected counterpart of the MOS-DH listed in Table 1.

d The D4 dispersion corrected counterpart of the xMOS-DH listed in Table 1.

Next, adding a dispersion correction to xMOS_78_-PBEP86 yields only a marginal improvement in accuracy (0.06 kcal mol^–1^). As before, most of this gain is due to the MCONF and PCONF21 subsets, while for the W4–11 subset, the inclusion of the D4 correction does more harm than good (Table S18 in the Supporting Information). In terms of WTMAD-2 for GMTKN55, the accuracy of this dispersion-corrected MOS-DH is comparable to that of xDOD_72_-PBEP86-D4. Further, optimizing the MOS-MP2 attenuation parameter (ω) and the exact exchange fraction (*a*_X_), we obtain the lowest WTMAD-2 of 2.19 kcal mol^–1^ for *a*_X_ = 0.78 and ω = 0.80 (Table S17 in the Supporting Information).

Hence, for the GMTKN55 benchmark, MOS double hybrids exhibit performance comparable to their SOS-MP2-based counterparts when the D4 dispersion correction is applied.

### 5.3 Transition Metal Reactions (TM9)

The TM9 set comprises barrier heights, as represented by LTMBH, MOBH35, and TMBH,^17,82,84–87^ closed- and open-shell organometallic reaction energies, as in ROST61, MOR41, and WCCR10,^18,19,90,91^ coinage metal clusters from CUAGAU-2,^81^ ionization potentials from TMIP,^89^ and conformational energies from TMCONF16.^88^ Total WTMAD-2_TM9_ and mean absolute errors for individual transition metal sets are provided in Table S24 in the Supporting Information.

For both, MOS_76_-PBEP86 and xMOS_78_-PBEP86 we find a very similar WTMAD-2_TM_ of 2.84 and 2.69 kcal mol^–1^, respectively. This is also reflected in the mean absolute errors of individual subsets (see Table 4). MOS_76_-PBEP86 is found to perform better on the ROST61 and WCCR10 sets, whereas xMOS_78_-PBEP86 performs better on the TMIP and CUAGAU-2.

**Table 4:** Total WTMAD-2 for the TM9 Set and Mean Absolute Errors for Nine Metal-Organic Benchmark Subsets in kcal mol^–1^ ^a^

**functional**	**WTMAD-2**_**TM9**_	CUAGAU-2	LTMBH	MOBH35	MOR41	ROST61	TMBH	TMCONF16	TMIP	WCCR10
MOS_76_-PBEP86	2.84	4.17	0.88	1.65	3.82	3.43	1.13	0.22	14.85	1.65
xMOS_78_-PBEP86	2.69	4.01	0.80	1.62	3.79	2.95	1.13	0.23	10.31	1.80
MOS_76_-PBEP86-D4	2.86	4.17	0.88	1.75	3.64	3.46	1.13	0.20	14.85	2.13
xMOS_78_-PBEP86-D4	2.71	4.01	0.80	1.73	3.61	3.03	1.14	0.20	10.30	2.53
revDOD-PBEP86-D4 ^14^	1.96	3.00	0.51	1.06	2.63	2.12	0.86	0.19	9.24	1.30
Pr^2^SCAN69-D4 ^22^	1.91	3.23	0.40	1.62	2.40	1.96	0.98	0.16	7.96	1.86

a The error statistics for Pr^2^SCAN69-D4 are taken from ref 22.

Unlike in main-group chemistry problems, adding dispersion correction does not significantly influence the overall WTMAD-2 for TM9 (see Table 4). Analysis of the individual subsets reveals that dispersion correction provides only marginal improvement for MOR41 and TMCONF16 reactions, while for WCCR10, it does more harm than good.

A comparison of both MOS double-hybrids with the non-MOS functionals revDOD-PBEP86-D4 or Pr^2^SCAN69-D4 reveals that MOS-DHs generally perform worse for nearly all transition-metal sets. The barrier-height sets are least affected, likely due to the large amounts of Fock exchange used in all four DHs. However, the higher exact exchange in MOS-DHs likely causes the performance deterioration for the other transition-metal sets. While a large amount of Fock exchange can sometimes be favorable for main-group thermochemistry – as observed for GMTKN55 – in can be problematic for transition-metal chemistry where a higher degree of static correlation effects can be expected. Open-shell transition-metal complexes in ROST61, CUAGAU-2, and TMIP are particularly susceptible to these effects.

Comparing the mean WTMAD-2 values for the GMTKN55 and TM9 data sets combined (i.e., WTMAD-2_Mean_), it is evident that xMOS_78_-PBEP86 is the best performer among the new MOS double hybrids. However, it still underperformed compared to regular SOS-MP2-based dispersion-corrected functionals, such as revDOD-PBEP86-D4 or Pr^2^SCAN69-D4 (Figure 4).

**Figure 4:**
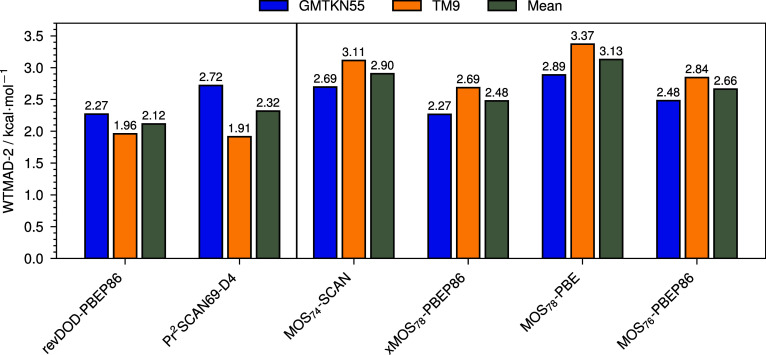
WTMAD-2 error statistics for GMTKN55, the TM9 transition metal sets, and their mean for MOS-double-hybrids, revDOD-PBEP86-D4, and Pr^2^SCAN69-D4. The results for Pr^2^SCAN69-D4 are extracted from ref 22.

## 6 Conclusions

We have proposed a new variety of double hybrid functionals using the modified opposite-spin MP2 as the nonlocal correlation component. From our investigation of MOS-DHs and their respective revDOD-family counterparts with the aid of the GMTKN55 and nine additional transition-metal data sets, we can conclude the following:

1. The MOS-MP2-based double hybrids always outperform their SCS-MP2-based counterparts on the GMTKN55 when any empirical dispersion correction is not used.
2. The key advantage of employing distance-dependent MP2 over regular MP2 in double hybrids originates from its improved handling of long-range correlation effects, which is particularly critical for inter- and intramolecular noncovalent interactions.
3. For a fixed value of HF-exchange in MOS-DHs, gradually increasing attenuation parameter (ω) requires a systematically smaller amount of MOS-MP2 correlation.
4. Among the MOS-DHs proposed in the present study, the four-parameter xMOS_78_-PBEP86 exhibits the best overall performance, effectively balancing the main-group and transition-metal chemistry performance.
5. Although MOS_74_-SCAN marginally outperforms Pr^2^SCAN69-D4 on the GMTKN55 benchmark, the latter functional significantly excels in reactions involving transition metals.
6. In terms of the WTMAD-2_Mean_ metric, all dispersion-uncorrected MOS double hybrids perform worse than Pr^2^SCAN69-D4 or revDOD-PBEP86-D4.
7. When D4 correction is incorporated into double hybrids, using the MOS-MP2 scheme does not provide additional benefits compared to simple SOS-MP2.

## Supplementary Material



## Data Availability

The data that support the findings of this study, including all energies and statistical analyses for the GMTKN55 benchmark data set calculated with the functionals presented in this work, are openly available in the GitHub repository at https://github.com/santra-compchem/MOS-DH. Additional details and files can be provided by the authors upon reasonable request.

## Notes

Description of the subsets of GMTKN55; a definition of the statistical measures; a comparison of MOS-MP2 vs SOS-MP2 in the DH framework; optimized parameters and WTMAD-2 for a few additional functionals; WTMAD-2 of different MOS- and xMOS-DHs; parameters of MOS-DHs for various %HFx andω in MOS-MP2; comparison of xDOD_n_-PNEP86-D4 and xMOS_n_-PBEP86; WTMAD-2 of different MOS- and xMOS-DHs imposing the *a*_C,DFA_ + *a*_OS_ = 1.0 constraint; TM9 results for MOS-DHs; and supplemental figures (PDF)
