# Supplementary material for: Modified Opposite-Spin-Scaled Double-Hybrid Functionals
Source: J Phys Chem A. 2025 Jul 24;129(31):7218–28. doi: 10.1021/acs.jpca.5c01035 (PMC12337148; doi:10.1021/acs.jpca.5c01035)
Supplement: Supplementary file 1 [file jp5c01035_si_001.pdf]

Supporting Information: Modified Opposite-Spin-Scaled Double-Hybrid Functionals

Golokesh Santra*,[1,2,a)] Markus Bursch*,[3,4,a)] and Lukas Wittmann*[4,a)]
[1)] *Max-Planck-Institut für Kohlenforschung, Kaiser-Wilhelm-Platz 1, D-45470 Mülheim an der Ruhr, Germany*
[2)] *Interdisciplinary Center for Scientific Computing, Ruprecht-Karls-Universität Heidelberg, Im Neuenheimer Feld 205, 69120 Heidelberg, Germany*
[3)] *FACCTs GmbH, 50677, Köln, Germany*
[4)] *Mulliken Center for Theoretical Chemistry, Universität Bonn, Beringstr. 4, 53115 Bonn, Germany*

(*Electronic mail: wittmann@thch.uni-bonn.de)

(*Electronic mail: bursch@kofo.mpg.de)

(*Electronic mail: santra@kofo.mpg.de; golokesh.santra@iwr.uni-heidelberg.de)

(Dated: 4 July 2025)

CONTENTS

A. Description of the 55 subsets of GMTKN55 S3

B. Definition of Statistical Measures S6

C. MOS-MP2 vs. SOS-MP2 in a double hybrid functional S8

D. Optimized parameters and performance statistics of a few additional functionals S12

E. WTMAD-2 of different MOS- and xMOS-DHs S13

F. Optimized parameters of MOS-DHs for different fractions of HF-exchange and range-separation parameters (ω) in MOS-MP2 S15

G. Comparing xDODn-PNEP86-D4 and xMOSn-PBEP86 S16

H. WTMAD-2 for different MOS-DH-D4 and xMOS-DH-D4s S17

I. WTMAD-2 of different MOS- and xMOS-DHs imposing the $a_{C,DFA} + a_{OS} = 1.0$ constraint during optimization. S20

J. TM results for MOS-DHs, revDSD-PBEP86-D4, revDOD-PBEP86-D4 and Pr^{2}SCAN69-D4 functionals S22
1. MOS$_{74}$-SCAN S22
2. xMOS$_{78}$-PBEP86 S22
3. MOS$_{78}$-PBE S22
4. MOS$_{76}$-PBEP86 S23
5. MOS$_{74}$-SCAN-D4 S23
6. xMOS$_{78}$-PBEP86-D4 S23
7. MOS$_{78}$-PBE-D4 S23
8. MOS$_{76}$-PBEP86-D4 S24

[a)] these authors contributed equally

K. Results for S66x8 S26

L. Figures S27

References S34

Appendix A: Description of the 55 subsets of GMTKN55

TABLE S1: Subsets of GMTKN55 with appropriate references. (basic= basic thermochemistry, barrier= barrier heights, large= large molecule involving reactions, intra= intramolecular noncovalent interactions, and inter= intermolecular noncovalent interactions)

Subcategory	Abbreviation	Description	Refs.
basic	AL2X6	Dimerization energies of AlX_3 compounds (X= H, F, Cl, Br, CH_3)	1
basic	ALK8	Dissociation and other reactions of alkaline compounds	1
basic	ALKBDE10	Dissociation energies in group-1 and -2 diatomics	2
basic	BH76RC	Reaction energies of the BH76[3–5] set	5
basic	DC13	Difficult cases for DFT methods	5–18
basic	DIPCS10	Double-ionization potentials of closed-shell systems	1
basic	FH51	Reaction energies in various (in-)organic systems	17,18
basic	G21EA	Adiabatic electron affinities	5,19
basic	G21IP	Adiabatic ionization potentials	5,19
basic	G2RC	Reaction energies of selected G2/97[20] systems	5,20
basic	HEAVYSB11	Dissociation energies in heavy-element compounds	1
basic	NBPRC	Oligomerizations and H_2 fragmentations of NH_3/BH_3 systems; H_2 activation reactions with PH_3/BH_3 systems	5,21,22
basic	PA26	Adiabatic proton affinities (incl. of amino acids)	1
basic	RC21	Fragmentations and rearrangements in radical cations	1
basic	SIE4x4	Self-interaction-error related problems	1
basic	TAUT15	Relative energies in tautomers	1
basic	W4-11	Total atomization energies	23
basic	YBDE18	Bond-dissociation energies in ylides	24
barrier	BH76	Barrier heights of hydrogen transfer, heavy atom transfer, nucleophilic substitution, unimolecular and association reactions	3–5
barrier	BHDIV10	Diverse reaction barrier heights	1
barrier	BHPERI	Barrier heights of pericyclic reactions	5,25–27
barrier	BHROT27	Barrier heights for rotation around single bonds	1
barrier	INV24	Inversion/racemization barrier heights	28
barrier	PX13	Proton-exchange barriers in H_2O, NH_3, and HF clusters	29

Continued on next page

TABLE S1: Subsets of GMTKN55 with appropriate references. (basic= basic thermochemistry, barrier= barrier heights, large= large molecule involving reactions, intra= intramolecular noncovalent interactions, and inter= intermolecular noncovalent interactions) (Continued)

Subcategory	Abbreviation	Description	Refs.
barrier	WCPT18	Proton-transfer barriers in uncatalysed and water-catalyzed reactions	30
large	BSR36	Bond-separation reactions of saturated hydrocarbons	31,32
large	C60ISO	Relative energies between C60 isomers	33
large	CDIE20	Double-bond isomerization energies in cyclic systems	34
large	DARC	Reaction energies of Diels–Alder reactions	5,35
large	ISO34	Isomerization energies of small and medium-sized organic molecules	36
large	ISOL24	Isomerization energies of large organic molecules	37
large	MB16-43	Decomposition energies of artificial molecules	1
large	PARel	Relative energies in protonated isomers	1
large	RSE43	Radical-stabilization energies	38
intra.	ACONF	Relative energies of alkane conformers	39
intra.	AMINO20x4	Relative energies in amino acid conformers	40
intra.	BUT14DIOL	Relative energies in butane-1,4-diol conformers	41
intra.	ICONF	Relative energies in conformers of inorganic systems	1
intra.	IDISP	Intramolecular dispersion interactions	5,21,36,42,43
intra.	MCONF	Relative energies in melatonin conformers	44
intra.	PCONF21	Relative energies in tri- and tetra-peptide conformers	45,46
intra.	SCONF	Relative energies of sugar conformers	5,47
intra.	UPU23	Relative energies between RNA-backbone conformers	48
inter.	ADIM6	Interaction energies of *n*-alkane dimers	49
inter.	AHB21	Interaction energies in anion–neutral dimers	50
inter.	CARBHB12	Hydrogen-bonded complexes between carbene analogues and H_2O, NH_3, or HCl	1
inter.	CHB6	Interaction energies in cation–neutral dimers	50
inter.	HAL59	Binding energies in halogenated dimers (incl. halogen bonds)	51,52
inter.	HEAVY28	Noncovalent interaction energies between heavy element hydrides	49
inter.	IL16	Interaction energies in anion–cation dimers	50
inter.	PNICO23	Interaction energies in pnicogen-containing dimers	53

Continued on next page

TABLE S1: Subsets of GMTKN55 with appropriate references. (basic= basic thermochemistry, barrier= barrier heights, large= large molecule involving reactions, intra= intramolecular noncovalent interactions, and inter= intermolecular noncovalent interactions) (Continued)

**Subcategory**	**Abbreviation**	**Description**	**Refs.**
inter.	RG18	Interaction energies in rare-gas complexes	1
inter.	S22	Binding energies of noncovalently bound dimers	54
inter.	S66	Binding energies of noncovalently bound dimers	55
inter.	WATER27	Binding energies in $(H_2O)n$, $H^+(H_2O)n$, and $OH^-(H_2O)n$	56

Appendix B: Definition of Statistical Measures

Error: The error e for a given interaction i is given by the difference of the actual method and the reference.

$$e_i = \text{method}_i - \text{reference}_i \tag{S1}$$

Mean Deviation (MD): Mean of the signed error, n corresponds to the number of data points of the respective set.

$$\text{MD} = \frac{1}{n}\sum_{i=1}^{n} e_i \equiv \bar{e} \tag{S2}$$

Mean Absolute Deviation (MAD): The term mean absolute deviation (MAD) is used in this work interchangeably with the **mean absolute** (unsigned) **error** (**MAE**).

$$\text{MAD} \overset{\text{this work}}{\equiv} \text{MAE} = \frac{1}{n}\sum_{i=1}^{n} |e_i| \tag{S3}$$

Root Mean Square Deviation (RMSD): The term root mean square deviation (RMSD) in this work is used interchangeably with **root mean squared error** (**RMSE**).

$$\text{RMSD} \overset{\text{this work}}{\equiv} \text{RMSE} = \sqrt{\frac{1}{n}\sum_{i=1}^{n} (e_i)^2} \tag{S4}$$

Standard Deviation (SD): The standard deviation is defined using Bessel's correction.

$$\text{SD} = \sqrt{\frac{1}{n-1}\sum_{i=1}^{n} (e_i - \bar{e})^2} \tag{S5}$$

Weighted Total Mean Absolute Deviation (WTMAD-2)
Of all N sets, every set j has n_j interactions, an average absolute interaction energy of $\overline{|\Delta E|}_j$ and mean absolute error MAD_j.

$$n_{\text{total}} = \sum_{j}^{N} n_j \qquad \overline{|\Delta E|}_{\text{total}} = \frac{1}{n_{\text{total}}}\sum_{j}^{N} \overline{|\Delta E|}_j$$

$$\text{WTMAD-2} = \sum_{j}^{N} \frac{n_j}{n_{\text{total}}} \frac{\overline{|\Delta E|}_{\text{total}}}{\overline{|\Delta E|}_j} \text{MAD}_j \tag{S6}$$

where $\overline{|\Delta E|}_{\text{total}}$ denotes the average absolute interaction energy of all N sets with n_{total} interactions. In the original GMTKN55 article, the reported $\overline{|\Delta E|}_{\text{total}}$ was 56.84.[1] However, the average absolute reaction energies for the NBPRC and MB16-43 subsets provided there differ from the corresponding values calculated from the individual data reported in the Supporting Information. Using the correct average absolute reaction energies, the $\overline{|\Delta E|}_{\text{total}}$ becomes 57.82 instead.

To be consistent with the previously reported WTMAD-2s of revised DSD functionals,[57] xDSD-PBEP86,[58,59] and XYG-based double hybrids,[60] we have used $\overline{|\Delta E|}_{\text{total}}$ = 56.84 in the present study. It goes without saying, however, that this will not affect the ranking of the functionals. As mentioned

in refs.[58,59], total WTMAD-$2_{56.84}$ can be converted to WTMAD-$2_{57.76}$ simply by multiplying the former value by 1.0162.

The 55 reaction sets in GMTKN55 are grouped into five major subcategories: (a) basic thermochemistry, (b) barrier heights, (c) reactions involving large molecules, (d) intramolecular noncovalent interactions, and (e) intermolecular noncovalent interactions. The contribution of each subcategory (or each of the 55 subsets) to the total WTMAD-2 is denoted as ΔWTMAD-2 and is given by:

$$\text{WTMAD-2}_{GMTKN55} = \sum_{j=1}^{5} \Delta\text{WTMAD-2}_j = \sum_{k=1}^{55} \Delta\text{WTMAD-2}_k \tag{S7}$$

Appendix C: MOS-MP2 vs. SOS-MP2 in a double hybrid functional

TABLE S2. Total WTMAD2, optimized PT2 parameters, and ΔWTMAD-2 contributions from the five major GMTKN55 subsets for the SOS-PT2 and MOS-PT2 based double hybrids.

Functionals	PT2 part	WTMAD-2 (kcal·mol^{-1})	Parameters		ΔWTMAD-2 (kcal·mol^{-1})				
			ω	a_{OS}	basic[a]	barrier[b]	large[c]	intra[d]	inter[e]
noDispOD-PBEP86[f]	SOS-PT2	4.02	—	0.6914	0.94	0.44	0.75	0.83	1.05
	MOS-PT2	2.77	0.40	0.4986	0.58	0.39	0.66	0.46	0.68
xnoDispOD-PBEP86[g]	SOS-PT2	3.53	—	0.7592	0.87	0.37	0.64	0.68	0.98
	MOS-PT2	2.36	0.37	0.5514	0.53	0.30	0.58	0.39	0.55

[a] basic thermochemistry; [b] barrier heights; [c] large molecule involving reactions; [d] intramolecular noncovalent interactions; [e] intermolecular noncovalent interactions;

[f] Parameters which were kept constant in both SOS-PT2 and MOS-PT2-based DHs: a_X= 0.69, $a_{X,DFA}$= 0.31, and $a_{C,DFA}$= 0.5340;

[g] Parameters which were kept constant in both SOS-PT2 and MOS-PT2-based xDHs: a_X= 0.72, $a_{X,DFA}$= 0.28, and $a_{C,DFA}$= 0.4742.

TABLE S3: ΔWTMAD-2 contributions from 55 subsets of GMTKN55 for the SOS-PT2 and MOS-PT2 based double hybrids.

Subsets	noDispOD$_{69}$-PBEP86		xnoDispOD$_{72}$-PBEP86	
	SOS-PT2	MOS-PT2	SOS-PT2	MOS-PT2
ACONF	0.042	0.026	0.039	0.026
ADIM6	0.085	0.048	0.075	0.037
AHB21	0.014	0.014	0.014	0.013
AL2X6	0.009	0.008	0.009	0.008
ALK8	0.007	0.004	0.007	0.004
ALKBDE10	0.016	0.014	0.014	0.020
AMINO20X4	0.164	0.122	0.145	0.112
BH76RC	0.061	0.062	0.058	0.062
BH76	0.243	0.190	0.218	0.168
BHDIV10	0.014	0.014	0.011	0.009
BHPERI	0.056	0.086	0.035	0.055
BHROT27	0.016	0.022	0.013	0.019
BSR36	0.236	0.146	0.198	0.117
BUT14DIOL	0.054	0.049	0.056	0.040
C60ISO	0.028	0.041	0.031	0.042
CARBHB12	0.033	0.036	0.027	0.028
CDIE20	0.073	0.100	0.062	0.092
CHB6	0.007	0.007	0.007	0.007
DARC	0.017	0.015	0.010	0.010
DC13	0.038	0.030	0.035	0.025

Continued on next page

TABLE S3: ΔWTMAD-2 contributions from 55 subsets of GMTKN55 for the SOS-PT2 and MOS-PT2 based double hybrids. (Continued)

Subsets	noDispOD$_{69}$-PBEP86		xnoDispOD$_{72}$-PBEP86	
	SOS-PT2	MOS-PT2	SOS-PT2	MOS-PT2
DIPCS10	0.001	0.001	0.001	0.001
FH51	0.070	0.054	0.061	0.043
G21EA	0.081	0.050	0.068	0.055
G21IP	0.017	0.010	0.014	0.009
G2RC	0.039	0.044	0.034	0.036
HAL59	0.171	0.146	0.154	0.127
HEAVY28	0.120	0.080	0.137	0.064
HEAVYSB11	0.012	0.006	0.010	0.006
ICONF	0.022	0.017	0.022	0.020
IDISP	0.023	0.014	0.019	0.013
IL16	0.003	0.002	0.002	0.002
INV24	0.019	0.015	0.018	0.015
ISO34	0.055	0.043	0.048	0.036
ISOL24	0.048	0.051	0.046	0.049
MB16-43	0.028	0.031	0.029	0.038
MCONF	0.102	0.042	0.071	0.057
NBPRC	0.008	0.005	0.006	0.004
PA26	0.007	0.006	0.008	0.007
PAREL	0.095	0.086	0.084	0.073
**PCONF21**	**0.319**	**0.120**	**0.231**	**0.062**
PNICO23	0.037	0.039	0.035	0.025
PX13	0.051	0.030	0.043	0.018
RC21	0.033	0.028	0.030	0.027
RG18	0.218	0.141	0.190	0.122
RSE43	0.173	0.149	0.133	0.125
S22	0.072	0.023	0.065	0.017
**S66**	**0.277**	**0.128**	**0.254**	**0.101**
SCONF	0.012	0.006	0.018	0.006
SIE4X4	0.110	0.084	0.100	0.070
TAUT15	0.118	0.082	0.114	0.072
UPU23	0.092	0.060	0.080	0.056
**W4-11**	**0.264**	**0.074**	**0.248**	**0.065**
WATER27	0.015	0.014	0.018	0.012
WCPT18	0.041	0.033	0.033	0.020
YBDE18	0.054	0.020	0.049	0.011

TABLE S4: ΔWTMAD-2 contributions from 55 subsets of GMTKN55.

Subset	revDOD-PBEP86-D4	MOS_{76}-PBEP86 (ω=0.50)	$xDOD_{72}$ PBEP86-D4	$xMOS_{78}$-PBEP86 (ω=0.65)	$noDispSD_{82}$-PBEP86	$xnoDispSD_{82}$-PBEP86
ACONF	0.008	0.020	0.011	0.018	0.034	0.034
ADIM6	0.018	0.042	0.021	0.037	0.058	0.048
AHB21	0.008	0.009	0.007	0.010	0.013	0.013
AL2X6	0.010	0.008	0.008	0.007	0.009	0.009
ALK8	0.010	0.005	0.008	0.005	0.009	0.009
ALKBDE10	0.011	0.020	0.011	0.026	0.012	0.011
AMINO20X4	0.151	0.121	0.152	0.108	0.146	0.131
BH76RC	0.047	0.066	0.049	0.072	0.057	0.049
BH76	0.154	0.206	0.152	0.193	0.255	0.203
BHDIV10	0.006	0.009	0.004	0.007	0.010	0.008
BHPERI	0.026	0.087	0.018	0.070	0.122	0.095
BHROT27	0.014	0.011	0.012	0.008	0.026	0.022
BSR36	0.123	0.086	0.104	0.064	0.038	0.033
BUT14DIOL	0.039	0.041	0.050	0.030	0.041	0.044
C60ISO	0.020	0.043	0.021	0.040	0.033	0.035
CARBHB12	0.025	0.024	0.021	0.021	0.028	0.025
CDIE20	0.057	0.077	0.053	0.063	0.086	0.081
CHB6	0.006	0.006	0.007	0.006	0.007	0.007
DARC	0.007	0.010	0.005	0.006	0.022	0.014
DC13	0.016	0.026	0.017	0.023	0.032	0.027
DIPCS10	0.002	0.001	0.002	0.002	0.002	0.003
FH51	0.050	0.041	0.046	0.041	0.066	0.060
G21EA	0.073	0.062	0.074	0.063	0.063	0.068
G21IP	0.012	0.011	0.011	0.010	0.009	0.009
G2RC	0.029	0.034	0.028	0.032	0.027	0.023
HAL59	0.106	0.116	0.119	0.107	0.119	0.113
HEAVY28	0.088	0.066	0.094	0.062	0.072	0.085
HEAVYSB11	0.010	0.008	0.008	0.007	0.004	0.003
ICONF	0.022	0.021	0.023	0.020	0.025	0.024
IDISP	0.009	0.010	0.010	0.009	0.017	0.014
IL16	0.002	0.003	0.001	0.002	0.003	0.002
INV24	0.020	0.017	0.020	0.017	0.021	0.020
ISO34	0.033	0.034	0.034	0.032	0.048	0.041
ISOL24	0.044	0.041	0.042	0.038	0.044	0.040
MB16-43	0.054	0.038	0.048	0.045	0.015	0.013
MCONF	0.033	0.066	0.029	0.067	0.062	0.072
NBPRC	0.004	0.004	0.003	0.005	0.003	0.003
PA26	0.008	0.005	0.009	0.004	0.005	0.005

Continued on next page

TABLE S4: ΔWTMAD-2 contributions from 55 subsets of GMTKN55. (Continued)

Subset	revDOD-PBEP86-D4	MOS_{76}-PBEP86 (ω=0.50)	$xDOD_{72}$ PBEP86-D4	$xMOS_{78}$-PBEP86 (ω=0.65)	$noDispSD_{82}$-PBEP86	$xnoDispSD_{82}$-PBEP86
PAREL	0.056	0.069	0.055	0.063	0.080	0.075
PCONF21	0.047	0.070	0.046	0.067	0.113	0.059
PNICO23	0.021	0.021	0.019	0.018	0.026	0.025
PX13	0.019	0.015	0.015	0.012	0.030	0.025
RC21	0.033	0.038	0.033	0.034	0.068	0.057
RG18	0.094	0.138	0.085	0.133	0.162	0.134
RSE43	0.187	0.242	0.151	0.185	0.287	0.200
S22	0.016	0.021	0.012	0.017	0.025	0.022
S66	0.075	0.093	0.075	0.090	0.142	0.129
SCONF	0.013	0.009	0.011	0.008	0.012	0.019
SIE4X4	0.088	0.051	0.079	0.044	0.044	0.048
TAUT15	0.114	0.048	0.124	0.039	0.058	0.058
UPU23	0.077	0.053	0.075	0.054	0.059	0.057
W4-11	0.048	0.076	0.049	0.093	0.066	0.051
WATER27	0.006	0.007	0.008	0.007	0.009	0.009
WCPT18	0.015	0.021	0.013	0.017	0.028	0.023
YBDE18	0.011	0.018	0.011	0.013	0.038	0.030

Appendix D: Optimized parameters and performance statistics of a few additional functionals

TABLE S5. Final parameters of a few MOS-DHs, noDispSD, revDSD, revDOD, and xDH functionals.[(a)]

Functionals	ω	a_X	$a_{X,DFA}$	$a_{C,DFA}$	a_{OS}	a_{SS}	s_6	a_1	a_2	s_8	s_9
MOS_{74}-SCAN	0.90	0.74	0.26	0.4938	0.4700	[0]	—	—	—	—	—
MOS_{78}-PBE	0.50	0.78	0.22	0.4407	0.6284	[0]	—	—	—	—	—
$noDispSD_{69}$-SCAN[(b)]	—	0.69	0.31	0.4472	0.6194	0.2533	—	—	—	—	—
$noDispSD_{68}$-PBE	—	0.84	0.16	0.2733	0.7822	0.4693	—	—	—	—	—
revDOD-PBEP86-D4[(c)]	—	0.69	0.31	0.4301	0.6131	[0]	0.6158	0.3440	4.2426	[0]	[1.0]
revDOD-SCAN-D4[(c)]	—	0.66	0.34	0.4780	0.6433	[0]	0.4088	0.2571	4.5012	[0]	[1.0]
revDOD-PBE-D4[(c)]	—	0.68	0.32	0.4465	0.6175	[0]	0.7410	0.3773	3.7696	[0]	[1.0]
$xDOD_{72}$-PBEP86-D4[(d)]	—	0.72	0.28	0.3999	0.6743	[0]	0.5395	0.2095	5.0154	[0]	[1.0]
revDSD-PBEP86-D4[(c)]	—	0.69	0.31	0.4224	0.5935	0.0566	0.5917	0.3710	4.2014	[0]	[1.0]
revDSD-PBE-D4[(c)]	—	0.68	0.32	0.4371	0.6111	0.0318	0.7226	0.3842	3.7561	[0]	[1.0]
$xDSD_{75}$-PBEP86-D4[(d)]	—	0.75	0.25	0.3524	0.6894	0.1260	0.4259	0.2822	4.7222	[0]	[1.0]

[(a)]The parameters in the squire bracket are kept constant during optimization; [(b)]from ref.[57] [(c)]from refs.[57,58]; [(d)]from refs.[58,59]; [(e)]from ref.[60] [(f)]from ref.[61]

TABLE S6. Total WTMAD-2 (kcal·mol^{-1}) and its division into five major subcategories of GMTKN55.

Functionals	WTMAD-2 (kcal·mol^{-1})	ΔWTMAD-2 (kcal·mol^{-1})				
		basic[(a)]	barriers[(b)]	large[(c)]	intra.[(d)]	inter.[(e)]
MOS_{74}-SCAN	2.70	0.58	0.45	0.66	0.42	0.59
MOS_{78}-PBE	2.89	0.57	0.45	0.69	0.53	0.65
$noDispSD_{69}$-SCAN[(a)]	2.93	0.57	0.48	0.67	0.48	0.74
$noDispSD_{68}$-PBE	3.47	0.68	0.63	0.79	0.59	0.78
revDOD-SCAN-D4[(f)]	2.48	0.59	0.35	0.60	0.41	0.52
revDOD-PBE-D4[(f)]	2.37	0.64	0.29	0.53	0.42	0.49
revDSD-PBEP86-D4[(f)]	2.25	0.55	0.26	0.57	0.41	0.46
revDSD-PBE-D4[(f)]	2.36	0.64	0.30	0.52	0.42	0.48
$xDSD_{75}$-PBEP86-D4[(g)]	2.12	0.51	0.25	0.49	0.41	0.46

[(a)]basic thermochemistry; [(b)]barrier heights; [(c)]large molecule involving reactions; [(d)]intramolecular noncovalent interactions; [(e)]intermolecular noncovalent interactions; [(f)]from refs.[57,58]; [(g)]from refs.[58,59]; [(h)]from ref.[60] [(j)]Because of the use of def2-QZVPPD basis sets for seven GMTKN55 subsets (WATER27, RG18, IL16, G21EA, AHB21, BH76, and BH76RC), the total WTMAD-2 is 0.09 kcal/mol lower than that reported in ref.[61]

Appendix E: WTMAD-2 of different MOS- and xMOS-DHs

TABLE S7. WTMAD-2$_{\text{GMTKN55}}$ (kcal·mol^{-1}) errors of MOS$_n$-PBEP86 for different percentages of HF exchange (i.e., $n = 100a_X$) and MOS-MP2 attenuation parameters (ω).

ω	n=66	n=69	n=74	n=76	n=78	n=82
0.20	3.47	3.20	2.88	2.82	2.80	2.90
0.40	2.91	2.68	2.50	2.49	2.52	2.71
0.45	2.94	2.69	2.50	2.482	2.50	2.67
0.50	—	2.73	2.51	**2.480**	2.49	2.64
0.55	—	2.78	2.52	2.485	2.483	2.60
0.60	3.10	2.83	2.54	2.50	2.484	2.57
0.65	3.16	2.89	2.57	2.51	2.49	2.56
0.80	3.35	3.05	2.69	2.60	2.55	2.56
1.00	3.56	3.26	2.87	2.76	2.68	2.63
1.20	3.71	3.41	3.01	2.89	2.81	2.74
∞	—	4.01	—	—	—	—
0.001	—	4.01	—	—	—	—

TABLE S8. WTMAD-2$_{\text{GMTKN55}}$ (kcal·mol^{-1}) errors of xMOS$_n$-PBEP86 for different percentages of HF exchange (i.e., $n = 100a_X$) and MOS-MP2 attenuation parameters (ω).

ω	n=50	n=69	n=72	n=75	n=78	n=82	n=85
0.20	5.35	2.95	2.69	2.52	2.45	2.54	—
0.30	4.95	2.52	2.35	2.29	2.33	2.54	—
0.35	4.89	2.47	2.33	2.28	2.33	2.56	—
0.37	4.88	2.47	2.33	2.28	2.33	2.56	—
0.40	4.89	2.47	2.33	2.28	2.33	2.55	2.81
0.43	—	2.49	2.34	2.28	2.32	2.53	—
0.45	4.93	2.50	2.35	2.28	2.31	2.51	—
0.60	5.11	2.63	2.42	2.31	2.270	2.37	2.58
0.65	—	2.69	2.45	2.32	**2.266**	2.34	—
0.70	—	2.74	2.49	2.34	2.272	2.32	2.48
0.77	—	2.82	2.55	2.38	2.29	2.31	2.43
0.80	5.33	2.85	2.58	2.40	2.31	2.31	2.42
0.90	5.41	2.95	2.67	2.46	2.35	2.32	2.41
1.00	5.48	3.03	2.75	2.53	2.39	2.35	2.41
1.20	—	3.18	2.89	2.66	2.50	2.34	2.46
∞	—	3.80	3.53	3.30	3.14	3.04	—
0.001	—	3.80	3.53	3.30	3.14	3.04	—

TABLE S9. WTMAD-2_{GMTKN55} (kcal·mol^{-1}) errors of MOS$_n$-SCAN for different percentages of HF exchange (i.e., $n = 100a_X$) and MOS-MP2 attenuation parameters (ω).

ω	n=66	n=69	n=74	n=79
0.20	2.87	2.84	2.84	2.94
0.40	2.80	2.79	2.85	3.00
0.60	2.78	2.75	2.76	2.85
0.80	2.78	2.72	2.701	2.74
0.90	2.79	2.73	**2.695**	2.72
1.00	2.82	2.75	2.700	2.72
1.20	2.88	2.81	2.74	2.75

TABLE S10. WTMAD-2_{GMTKN55} (kcal·mol^{-1}) errors of MOS$_n$-PBE for different percentages of HF exchange (i.e., $n = 100a_X$) and MOS-MP2 attenuation parameters (ω).

ω	n=68	n=72	n=75	n=78	n=82
0.20	4.09	3.75	3.56	3.46	3.46
0.40	3.27	3.00	2.90	2.90	3.02
0.50	3.35	3.04	2.91	**2.886**	2.97
0.60	3.51	3.16	2.99	2.93	2.96
0.80	3.85	3.46	3.24	3.11	3.06
1.00	4.13	3.73	3.49	3.32	3.22
1.20	4.33	3.94	3.69	3.50	3.38

Appendix F: Optimized parameters of MOS-DHs for different fractions of HF-exchange and range-separation parameters (ω) in MOS-MP2

TABLE S11. Optimized semilocal and nonlocal correlation parameters of MOS$_n$-PBEP86 for different percentages of HF exchange (i.e., $n = 100a_X$) and MOS-MP2 attenuation parameters (ω).

	$a_{C,DFA}$						a_{OS}					
ω	n=66	n=69	n=74	n=76	n=78	n=82	n=66	n=69	n=74	n=76	n=78	n=82
0.20	0.5359	0.5150	0.4710	0.4543	0.4421	0.4045	0.6080	0.6458	0.7180	0.7429	0.7622	0.8288
0.40	0.5101	0.4887	0.4518	0.4352	0.4197	0.3869	0.5039	0.5316	0.5781	0.5766	0.5959	0.6426
0.45	0.5089	0.4892	0.4505	0.4357	0.4210	0.3836	0.4864	0.5145	0.5609	0.5975	0.6196	0.6640
0.50	—	0.4866	0.4529	0.4371	0.4227	0.3826	—	0.5023	0.5437	0.5602	0.5787	0.6247
0.55	—	0.4864	0.4526	0.4365	0.4248	0.3858	—	0.4905	0.5313	0.5492	0.5634	0.6070
0.60	0.5173	0.4887	0.4554	0.4383	0.4272	0.3874	0.4447	0.4783	0.5172	0.5373	0.5512	0.5937
0.65	0.5195	0.4923	0.4557	0.4373	0.4301	0.3937	0.4355	0.4671	0.5101	0.5297	0.5394	0.5787
0.80	0.5306	0.5026	0.4628	0.4442	0.4319	0.4033	0.4114	0.4405	0.4871	0.5032	0.5179	0.5507
1.00	0.5409	0.5127	0.4670	0.4516	0.4354	0.4096	0.3860	0.4156	0.4645	0.4830	0.4974	0.5278
1.20	0.5481	0.5212	0.4745	0.4567	0.4399	0.4116	0.3684	0.3971	0.4451	0.4648	0.4833	0.5117

TABLE S12. Optimized semilocal and nonlocal correlation parameters of xMOS$_n$-PBEP86 for different percentages of HF exchange (i.e., $n = 100a_X$) and MOS-MP2 attenuation parameters (ω).

	$a_{C,DFA}$							a_{OS}						
ω	n=50	n=69	n=72	n=75	n=78	n=82	n=85	n=50	n=69	n=72	n=75	n=78	n=82	n=85
0.20	0.6485	0.4855	0.4600	0.4371	0.4152	0.3776	—	0.4003	0.6605	0.7018	0.7378	0.7729	0.8343	—
0.30	0.6269	0.4728	0.4532	0.4291	0.4088	0.3697	—	0.3786	0.5823	0.6089	0.6454	0.6762	0.7333	—
0.35	0.6221	0.4666	0.4511	0.4284	0.4040	0.3746	—	0.3620	0.5557	0.5777	0.6108	0.6432	0.6905	—
0.37	0.6204	0.4670	0.4496	0.4278	0.4042	0.3737	—	0.3560	0.5446	0.5687	0.5994	0.6310	0.6788	—
0.40	0.6187	0.4688	0.4484	0.4288	0.4046	0.3726	0.3513	0.3489	0.5297	0.5560	0.5828	0.6150	0.6612	0.6923
0.43	—	0.4689	0.4494	0.4271	0.4040	0.3736	—	—	0.5178	0.5421	0.5717	0.6008	0.6437	—
0.45	0.6213	0.4691	0.4507	0.4275	0.4037	0.3725	—	0.3345	0.5111	0.5338	0.5633	0.5934	0.6352	—
0.60	0.6296	0.4671	0.4493	0.4296	0.4066	0.3699	0.3464	0.2994	0.4781	0.4965	0.5195	0.5455	0.5870	0.6176
0.65	—	0.4703	0.4520	0.4300	0.4056	0.3714	—	—	0.4690	0.4873	0.5110	0.5373	0.5770	—
0.70	—	0.4752	0.4521	0.4293	0.4064	0.3720	0.3479	—	0.4584	0.4806	0.5049	0.5284	0.5668	0.5941
0.77	—	0.4791	0.4536	0.4301	0.4092	0.3758	0.3513	—	0.4438	0.4731	0.4954	0.5176	0.5536	0.5817
0.80	0.6460	0.4805	0.4531	0.4319	0.4084	0.3777	0.3522	0.2629	0.4398	0.4704	0.4903	0.5143	0.5480	0.5770
0.90	0.6540	0.4869	0.4556	0.4357	0.4102	0.3750	0.3532	0.2508	0.4250	0.4586	0.4791	0.5019	0.5395	0.5644
1.00	0.6559	0.4900	0.4609	0.4376	0.4130	0.3780	0.3528	0.2435	0.4155	0.4452	0.4704	0.4914	0.5269	0.5546
1.20	—	0.4974	0.4658	0.4408	0.4193	0.3785	0.3528	—	0.3997	0.4282	0.4562	0.4770	0.5256	0.5420

Appendix G: Comparing xDODn-PNEP86-D4 and xMOSn-PBEP86

TABLE S13. WTMAD-2_{GMTKN55} (kcal·mol^{-1}) errors and optimized parameters of seven xMOS$_n$-PBEP86 functionals.

Functional	WTMAD-2	Parameters			
	(kcal·mol^{-1})	a_X	ω	$a_{C,DFA}$	a_{OS}
xMOS$_{50}$-PBEP86	4.88	0.50	0.37	0.6204	0.3560
xMOS$_{69}$-PBEP86	2.47	0.69	0.37	0.4670	0.5446
xMOS$_{72}$-PBEP86	2.33	0.72	0.37	0.4496	0.5687
xMOS$_{75}$-PBEP86	2.28	0.75	0.37	0.4278	0.5994
xMOS$_{78}$-PBEP86	2.27	0.78	0.65	0.4056	0.5373
xMOS$_{82}$-PBEP86	2.31	0.82	0.80	0.3777	0.5480
xMOS$_{85}$-PBEP86	2.41	0.85	0.90	0.3532	0.5644

TABLE S14. WTMAD-2_{GMTKN55} (kcal·mol^{-1}) errors and optimized parameters of seven xDOD$_n$-PBEP86-D4 functionals.

Functional	WTMAD-2	Parameters[(a)]						
	(kcal·mol^{-1})	a_X	$a_{C,DFA}$	a_{OS}	a_{SS}	s_6	a_1	a_2
xDOD$_{50}$-PBEP86-D4	3.29	0.50	0.5560	0.3014	[0]	0.9583	0.3083	4.8550
xDOD$_{69}$-PBEP86-D4	2.23	0.69	0.4316	0.6164	[0]	0.5909	0.2821	4.7998
xDOD$_{72}$-PBEP86-D4	2.20	0.72	0.3999	0.6743	[0]	0.5395	0.2095	5.0154
xDOD$_{75}$-PBEP86-D4	2.21	0.75	0.3694	0.7375	[0]	0.4864	0.1974	4.9580
xDOD$_{78}$-PBEP86-D4	2.27	0.78	0.3390	0.8026	[0]	0.4443	0.2096	4.7534
xDOD$_{82}$-PBEP86-D4	2.41	0.82	0.3031	0.8859	[0]	0.3932	0.2900	4.2001
xDOD$_{85}$-PBEP86-D4	2.56	0.85	0.2829	0.9469	[0]	0.3492	0.2822	4.2234

[(a)] $s_8 = 0$ and $s_9 = 1.0$ across the board.

TABLE S15. Contribution from the five major subcategories to the total WTMAD-2 (i.e., ΔWTMAD-2_{GMTKN55}) of xMOS$_n$-PBEP86 and xDOD$_n$-PBEP86-D4 functionals.

%HFx	xMOS$_n$-PBEP86					xDOD$_n$-PBEP86-D4				
(i.e., $n = 100a_X$)	basic[(a)]	barriers[(b)]	large[(c)]	intra.[(d)]	inter.[(e)]	basic[(a)]	barriers[(b)]	large[(c)]	intra.[(d)]	inter.[(e)]
50	0.91	0.87	1.07	1.03	0.99	0.93	0.42	0.81	0.58	0.54
69	0.55	0.34	0.62	0.41	0.54	0.58	0.23	0.53	0.42	0.46
72	0.53	0.30	0.59	0.40	0.51	0.57	0.23	0.51	0.41	0.47
75	0.51	0.29	0.57	0.43	0.48	0.58	0.25	0.51	0.40	0.47
78	0.52	0.32	0.53	0.38	0.51	0.60	0.28	0.50	0.40	0.48
82	0.52	0.34	0.53	0.41	0.51	0.63	0.35	0.53	0.41	0.49
85	0.54	0.36	0.55	0.45	0.51	0.66	0.41	0.57	0.42	0.50

[(a)]basic thermochemistry; [(b)]barrier heights; [(c)]large molecule involving reactions; [(d)]intramolecular noncovalent interactions; [(e)]intermolecular noncovalent interactions.

Appendix H: WTMAD-2 for different MOS-DH-D4 and xMOS-DH-D4s

TABLE S16. WTMAD-2_{GMTKN55} (kcal·mol^{-1}) errors of MOS$_n$-PBEP86-D4 for different percentages of HF exchange (i.e., $n = 100a_X$) and MOS-MP2 attenuation parameters (ω).

ω	n=69	n=74	n=76	n=78
0.10	2.27	—	—	—
0.20	2.31	2.34	2.38	2.45
0.40	2.42	2.36	2.37	2.41
0.50	2.43	2.36	2.38	2.38
0.60	2.42	2.35	2.35	2.36
0.80	2.39	2.31	2.31	2.32

TABLE S17. WTMAD-2_{GMTKN55} (kcal·mol^{-1}) errors of xMOS$_n$-PBEP86-D4 for different percentages of HF exchange (i.e., $n = 100a_X$) and MOS-MP2 attenuation parameters (ω).

ω	n=69	n=72	n=75	n=78
0.20	2.24	2.21	2.21	2.27
0.37	2.33	2.26	2.22	2.22
0.45	2.36	2.28	2.23	2.22
0.60	2.38	2.29	2.24	2.22
0.65	2.37	2.29	2.24	2.21
0.80	2.35	2.27	2.21	2.19

TABLE S18: ΔWTMAD-2 contributions from 55 subsets of GMTKN55 for dispersion corrected and uncorrected MOS-DHs

Subset	MOS_{76}-PBEP86 (ω=0.50)	$xMOS_{78}$-PBEP86 (ω=0.65)	MOS_{76}-PBEP86-D4 (ω=0.50)	MOS_{78}-PBEP86-D4 (ω=0.65)
ACONF	0.020	0.018	0.022	0.017
ADIM6	0.042	0.037	0.017	0.030
AHB21	0.009	0.010	0.009	0.010
AL2X6	0.008	0.007	0.009	0.007
ALK8	0.005	0.005	0.006	0.005
ALKBDE10	0.020	0.026	0.023	0.027
AMINO20X4	0.121	0.108	0.125	0.112
BH76RC	0.066	0.072	0.068	0.072
BH76	0.206	0.193	0.205	0.192
BHDIV10	0.009	0.007	0.008	0.007
BHPERI	0.087	0.070	0.084	0.072
BHROT27	0.011	0.008	0.010	0.008
BSR36	0.086	0.064	0.073	0.048
BUT14DIOL	0.041	0.030	0.044	0.031
C60ISO	0.043	0.040	0.042	0.041
CARBHB12	0.024	0.021	0.026	0.023
CDIE20	0.077	0.063	0.075	0.061
CHB6	0.006	0.006	0.005	0.006
DARC	0.010	0.006	0.009	0.006
DC13	0.026	0.023	0.023	0.023
DIPCS10	0.001	0.002	0.002	0.002
FH51	0.041	0.041	0.040	0.041
G21EA	0.062	0.063	0.066	0.066
G21IP	0.011	0.010	0.011	0.011
G2RC	0.034	0.032	0.033	0.033
HAL59	0.116	0.107	0.106	0.108
HEAVY28	0.066	0.062	0.059	0.064
HEAVYSB11	0.008	0.007	0.007	0.007
ICONF	0.021	0.020	0.022	0.020
IDISP	0.010	0.009	0.009	0.010
IL16	0.003	0.002	0.002	0.001
INV24	0.017	0.017	0.018	0.018
ISO34	0.034	0.032	0.033	0.031
ISOL24	0.041	0.038	0.046	0.042
MB16-43	0.038	0.045	0.045	0.046
MCONF	0.066	0.067	0.037	0.033
NBPRC	0.004	0.005	0.005	0.004

Continued on next page

TABLE S18: ΔWTMAD-2 contributions from 55 subsets of GMTKN55 for dispersion corrected and uncorrected MOS-DHs (Continued)

Subset	MOS_{76}-PBEP86 (ω=0.50)	$xMOS_{78}$-PBEP86 (ω=0.65)	MOS_{76}-PBEP86-D4 (ω=0.50)	MOS_{78}-PBEP86-D4 (ω=0.65)
PA26	0.005	0.004	0.005	0.004
PAREL	0.069	0.063	0.063	0.062
PCONF21	0.070	0.067	0.049	0.048
PNICO23	0.021	0.018	0.022	0.021
PX13	0.015	0.012	0.012	0.011
RC21	0.038	0.034	0.039	0.034
RG18	0.138	0.133	0.109	0.116
RSE43	0.242	0.185	0.251	0.190
S22	0.021	0.017	0.025	0.016
S66	0.093	0.090	0.088	0.085
SCONF	0.009	0.008	0.013	0.008
SIE4X4	0.051	0.044	0.049	0.044
TAUT15	0.048	0.039	0.043	0.037
UPU23	0.053	0.054	0.061	0.062
W4-11	0.076	0.093	0.092	0.104
WATER27	0.007	0.007	0.005	0.008
WCPT18	0.021	0.017	0.018	0.017
YBDE18	0.018	0.013	0.013	0.012

Appendix I: WTMAD-2 of different MOS- and xMOS-DHs imposing the $a_{C,DFA} + a_{OS} = 1.0$ constraint during optimization.

TABLE S19. Optimized parameters, total WTMAD-2_{GMTKN55}, and ΔWTMAD-2_{GMTKN55} for the PBEP86, SCAN, and PBE-based MOS-double-hybrids employing the $a_{C,DFA} + a_{OS} = 1.0$ constraint.

Functional	WTMAD-2	Parameters				ΔWTMAD-2 (kcal·mol^{-1})				
	(kcal·mol^{-1})	ω	a_X	$a_{C,DFA}$	a_{OS}	basic[(a)]	barriers[(b)]	large[(c)]	intra.[(d)]	inter.[(e)]
MOS_{76}-PBEP86	2.48	0.50	0.76	0.4403	0.5597	0.52	0.37	0.64	0.41	0.55
MOS_{74}-SCAN	2.74	0.70	0.74	0.5139	0.4861	0.61	0.46	0.66	0.45	0.56
MOS_{78}-PBE	3.04	0.65	0.78	0.4001	0.5999	0.58	0.46	0.68	0.54	0.78
xMOS_{75}-PBEP86	2.28	0.43	0.75	0.4281	0.5719	0.52	0.30	0.56	0.41	0.50

[(a)]basic thermochemistry; [(b)]barrier heights; [(c)]large molecule involving reactions; [(d)]intramolecular noncovalent interactions; [(e)]intermolecular noncovalent interactions

TABLE S20. WTMAD-2_{GMTKN55} (kcal·mol^{-1}) errors of MOS_n-PBEP86 for different percentages of HF exchange (i.e., $n = 100a_X$) and MOS-MP2 attenuation parameters (ω). During optimization the $a_{C,DFA} + a_{OS} =$ 1.0 constraint was used.

ω	n=69	n=74	n=76	n=78	n=82
0.10	6.10	—	—	—	—
0.20	4.92	5.33	5.54	5.76	6.25
0.40	2.74	2.61	2.65	2.74	3.05
0.45	2.69	2.51	2.50	2.54	2.78
0.50	2.75	2.51	**2.481**	2.49	2.64
0.55	2.86	2.56	2.52	2.51	2.61
0.60	2.97	2.65	2.60	2.57	2.62
0.65	3.08	2.76	2.69	2.65	2.66
0.80	3.39	3.05	2.96	2.91	2.88
1.00	3.71	3.36	3.27	3.20	3.14
1.20	3.95	3.60	3.50	3.43	3.37
∞	4.87	—	—	—	—
0.001	6.26	—	—	—	—

TABLE S21. WTMAD-2_{GMTKN55} (kcal·mol^{-1}) values of xMOS$_n$-PBEP86 for different percentages of HF exchange (i.e., $n = 100a_X$) and MOS-MP2 attenuation parameters (ω). During optimization the $a_{C,DFA} + a_{OS} =$ 1.0 constraint was used.

ω	n=50	n=69	n=72	n=75	n=78	n=82	n=85
0.20	5.46	4.50	4.68	4.93	5.23	5.68	—
0.30	4.95	2.94	2.94	3.06	3.27	3.66	—
0.35	4.90	2.57	2.48	2.52	2.69	3.04	—
0.37	4.92	2.50	2.39	2.40	2.54	2.87	—
0.40	4.96	2.47	2.34	2.31	2.39	2.69	2.99
0.43	—	2.52	2.35	**2.284**	2.32	2.57	—
0.45	5.05	2.56	2.39	2.30	2.31	2.52	—
0.60	5.36	2.94	2.75	2.64	2.60	2.64	2.77
0.65	—	3.07	2.88	2.75	2.70	2.73	—
0.70	—	3.19	2.99	2.86	2.80	2.81	2.90
0.77	—	3.34	3.13	2.99	2.93	2.93	3.00
0.80	5.68	3.40	3.19	3.05	2.98	2.97	3.04
0.90	5.80	3.57	3.36	3.21	3.12	3.11	3.17
1.00	5.89	3.72	3.51	3.35	3.25	3.23	3.29
1.20	—	3.96	3.75	3.58	3.47	3.22	3.49
∞	—	4.88	4.72	4.60	4.52	4.48	—
0.001	—	5.90	6.17	6.48	6.82	7.32	—

TABLE S22. WTMAD-2_{GMTKN55} (kcal·mol^{-1}) values of MOS$_n$-SCAN for different percentages of HF exchange (i.e., $n = 100a_X$) and MOS-MP2 attenuation parameters (ω). During optimization the $a_{C,DFA} + a_{OS} =$ 1.0 constraint was used.

ω	n=66	n=69	n=74	n=79
0.20	4.88	5.16	5.70	6.29
0.40	2.93	2.96	3.10	3.38
0.60	2.80	2.75	2.76	2.85
0.70	2.84	2.77	**2.742**	2.78
0.80	2.90	2.82	2.76	2.78
0.90	2.98	2.89	2.81	2.80
1.00	3.06	2.96	2.86	2.84
1.20	3.20	3.10	2.98	2.95

TABLE S23. WTMAD-2_{GMTKN55} (kcal·mol^{-1}) values of MOS$_n$-PBE for different percentages of HF exchange (i.e., $n = 100a_X$) and MOS-MP2 attenuation parameters (ω). During optimization the $a_{C,DFA} + a_{OS} =$ 1.0 constraint was used.

ω	n=68	n=72	n=75	n=78	n=82
0.20	6.67	6.86	7.06	7.28	7.60
0.40	3.92	3.76	3.74	3.80	3.98
0.50	3.64	3.36	3.26	3.25	3.37
0.60	3.63	3.29	3.13	3.07	3.12
0.65	—	3.32	3.13	**3.045**	3.07
0.80	3.87	3.48	3.26	3.12	3.07
1.00	4.13	3.73	3.49	3.32	3.22
1.20	4.33	3.95	3.70	3.51	3.38

Appendix J: TM results for MOS-DHs, revDSD-PBEP86-D4, revDOD-PBEP86-D4 and Pr^{2}SCAN69-D4 functionals

1. MOS$_{74}$-SCAN

Benchmark	ME	MAE	RMSE	SD	max	min	N_{set}	$\|E\|$
CUAGAU-2	-1.92	3.93	7.43	7.21	48.29	-16.70	123	78.27
LTMBH	-0.89	1.16	1.49	1.25	3.42	-0.70	13	8.23
MOBH35	-0.72	1.86	2.49	2.40	7.68	-4.06	70	20.89
MOR41	1.40	4.12	5.67	5.57	9.78	-21.83	41	31.20
ROST61	1.54	4.20	7.34	7.23	8.21	-41.99	61	42.78
TMBH	-0.75	1.29	1.67	1.52	3.56	-3.54	34	14.01
TMCONF16	0.02	0.21	0.26	0.27	0.70	-0.38	16	3.15
TMIP	7.25	15.40	26.89	27.16	22.73	-82.66	11	95.62
WCCR10	1.33	2.23	2.85	2.67	1.59	-5.47	9	48.27

2. xMOS$_{78}$-PBEP86

Benchmark	ME	MAE	RMSE	SD	max	min	N_{set}	$\|E\|$
CUAGAU-2	-2.50	4.01	7.01	6.57	48.56	-14.15	123	78.27
LTMBH	-0.65	0.80	1.02	0.82	2.24	-0.49	13	8.23
MOBH35	-0.55	1.62	2.29	2.24	7.61	-4.13	70	20.89
MOR41	1.35	3.79	5.26	5.15	7.76	-22.13	41	31.20
ROST61	0.50	2.95	4.34	4.35	13.63	-14.24	61	42.78
TMBH	-0.40	1.13	1.50	1.46	2.65	-4.65	34	14.01
TMCONF16	-0.03	0.23	0.27	0.28	0.70	-0.30	16	3.15
TMIP	-2.51	10.31	12.36	12.70	20.99	-19.71	11	95.62
WCCR10	1.28	1.80	2.70	2.52	1.34	-5.79	9	48.27

3. MOS$_{78}$-PBE

Benchmark	ME	MAE	RMSE	SD	max	min	N_{set}	$\|E\|$
CUAGAU-2	-1.82	4.52	7.96	7.78	46.81	-26.79	123	78.27
LTMBH	-0.78	1.19	1.50	1.33	3.22	-1.05	13	8.23
MOBH35	-0.89	2.21	2.97	2.86	8.92	-4.58	70	20.89
MOR41	1.68	4.51	6.43	6.28	9.50	-25.92	41	31.20
ROST61	1.37	3.97	6.79	6.71	10.67	-37.77	61	42.78
TMBH	-0.84	1.46	1.88	1.71	3.94	-4.44	34	14.01
TMCONF16	-0.02	0.18	0.24	0.24	0.63	-0.39	16	3.15
TMIP	6.58	15.39	26.50	26.92	20.73	-81.47	11	95.62
WCCR10	1.36	2.20	3.23	3.10	2.19	-6.79	9	48.27

4. MOS_{76}-PBEP86

Benchmark	ME	MAE	RMSE	SD	max	min	N_{set}	$\|E\|$
CUAGAU-2	-2.28	4.17	7.63	7.31	47.24	-16.74	123	78.27
LTMBH	-0.67	0.88	1.07	0.87	2.32	-0.68	13	8.23
MOBH35	-0.61	1.65	2.28	2.22	7.49	-4.06	70	20.89
MOR41	1.80	3.82	5.35	5.10	7.35	-22.84	41	31.20
ROST61	1.48	3.43	5.92	5.78	7.83	-32.62	61	42.78
TMBH	-0.46	1.13	1.48	1.42	2.89	-4.33	34	14.01
TMCONF16	-0.04	0.22	0.26	0.27	0.71	-0.29	16	3.15
TMIP	7.58	14.85	26.39	26.51	20.14	-81.69	11	95.62
WCCR10	0.92	1.65	2.39	2.34	1.50	-5.09	9	48.27

5. MOS_{74}-SCAN-D4

Benchmark	ME	MAE	RMSE	SD	max	min	N_{set}	$\|E\|$
CUAGAU-2	-1.91	3.93	7.43	7.21	48.28	-16.77	123	78.27
LTMBH	-0.89	1.16	1.49	1.24	3.42	-0.69	13	8.23
MOBH35	-0.81	2.00	2.80	2.70	9.42	-4.31	70	20.89
MOR41	0.98	3.94	5.58	5.56	9.84	-21.91	41	31.20
ROST61	1.25	4.24	7.37	7.33	9.24	-41.97	61	42.78
TMBH	-0.78	1.29	1.68	1.51	3.58	-3.53	34	14.01
TMCONF16	-0.00	0.18	0.21	0.22	0.58	-0.26	16	3.15
TMIP	7.28	15.40	26.90	27.15	22.72	-82.66	11	95.62
WCCR10	2.49	2.78	3.40	2.46	1.30	-6.48	9	48.27

6. $xMOS_{78}$-PBEP86-D4

Benchmark	ME	MAE	RMSE	SD	max	min	N_{set}	$\|E\|$
CUAGAU-2	-2.49	4.01	7.01	6.57	48.55	-14.21	123	78.27
LTMBH	-0.65	0.80	1.02	0.82	2.24	-0.49	13	8.23
MOBH35	-0.63	1.73	2.56	2.50	9.07	-4.18	70	20.89
MOR41	1.00	3.61	5.19	5.16	7.80	-22.20	41	31.20
ROST61	0.26	3.03	4.41	4.44	13.65	-14.42	61	42.78
TMBH	-0.42	1.14	1.49	1.45	2.67	-4.64	34	14.01
TMCONF16	-0.05	0.20	0.24	0.24	0.60	-0.22	16	3.15
TMIP	-2.48	10.30	12.36	12.70	20.98	-19.74	11	95.62
WCCR10	2.26	2.53	3.27	2.50	1.18	-6.84	9	48.27

7. MOS_{78}-PBE-D4

Benchmark	ME	MAE	RMSE	SD	max	min	N_{set}	$\|E\|$
CUAGAU-2	-1.81	4.52	7.97	7.79	46.80	-26.86	123	78.27
LTMBH	-0.79	1.20	1.50	1.33	3.22	-1.04	13	8.23
MOBH35	-0.98	2.35	3.29	3.16	10.68	-5.17	70	20.89
MOR41	1.26	4.42	6.37	6.33	9.56	-26.01	41	31.20
ROST61	1.07	4.00	6.86	6.84	11.72	-37.75	61	42.78
TMBH	-0.87	1.46	1.89	1.70	3.94	-4.43	34	14.01
TMCONF16	-0.04	0.16	0.20	0.20	0.52	-0.27	16	3.15
TMIP	6.62	15.39	26.50	26.91	20.73	-81.47	11	95.62
WCCR10	2.54	2.98	3.86	3.08	1.99	-8.03	9	48.27

8. MOS_{76}-PBEP86-D4

Benchmark	ME	MAE	RMSE	SD	max	min	N_{set}	$\|E\|$
CUAGAU-2	-2.27	4.17	7.63	7.31	47.23	-16.79	123	78.27
LTMBH	-0.67	0.88	1.07	0.87	2.32	-0.67	13	8.23
MOBH35	-0.68	1.75	2.53	2.46	8.85	-4.11	70	20.89
MOR41	1.47	3.64	5.26	5.11	7.39	-22.91	41	31.20
ROST61	1.25	3.46	5.95	5.87	8.62	-32.61	61	42.78
TMBH	-0.49	1.13	1.48	1.41	2.90	-4.32	34	14.01
TMCONF16	-0.06	0.20	0.24	0.24	0.62	-0.21	16	3.15
TMIP	7.61	14.85	26.39	26.50	20.13	-81.70	11	95.62
WCCR10	1.83	2.13	2.84	2.31	1.35	-6.07	9	48.27

TABLE S24. Total WTMAD-2 for TM9 and mean absolute errors for each of the nine metal-organic benchmark sets. The revDSD-PBEP86-D4 and Pr^{2}SCAN69-D4 error statistics are extracted from reference.[61]

	Data set	revDSD-PBEP86-D4	revDOD-PBEP86-D4	Pr^{2}SCAN69-D4	MOS$_{76}$-PBEP86	xMOS$_{78}$-PBEP86	MOS$_{74}$-SCAN	MOS$_{78}$-PBE
	CUAGAU-2	2.89	3.00	3.23	4.17	4.01	3.93	4.52
	LTMBH	0.57	0.51	0.40	0.88	0.80	1.16	1.19
	MOBH35	1.42	1.14	1.62	1.65	1.62	1.86	2.21
	MOR41	2.81	2.63	2.40	3.82	3.79	4.12	4.51
MAE	*ROST61*	2.04	2.12	1.96	3.43	2.95	4.20	3.97
(kcal·mol^{-1})	*TMBH*	0.84	0.86	0.98	1.13	1.13	1.29	1.46
	TMCONF16	0.18	0.19	0.16	0.22	0.23	0.21	0.18
	TMIP	9.01	9.23	7.96	14.85	10.31	15.40	15.39
	WCCR10	1.45	1.30	1.86	1.65	1.80	2.23	1.80
**WTMAD-2 (kcal·mol^{-1})**	***TM9***	**1.98**	**1.96**	**1.91**	**2.84**	**2.69**	**3.11**	**3.37**

Appendix K: Results for S66x8

TABLE S25. Root-mean-square deviations (in kcal/mol) for both dispersion-uncorrected and dispersion-corrected MOS-MP2- and SCS-MP2-based double hybrids are reported for the S66x8 benchmark. The reference interaction energies for S66x8 are at the "sterling silver" level.[62])

Functional	Full	Subsets											
	S66x8	H bonds	π–stack	London	Mixed	0.90r$_e$	0.95r$_e$	1.00r$_e$	1.05r$_e$	1.10r$_e$	1.25r$_e$	1.50r$_e$	2.00r$_e$
MOS$_{76}$-PBEP86	0.27	0.28	0.23	0.39	0.16	0.41	0.35	0.31	0.28	0.25	0.19	0.13	0.05
xMOS$_{78}$-PBEP86	0.23	0.23	0.17	0.35	0.14	0.34	0.29	0.26	0.23	0.21	0.18	0.14	0.06
MOS$_{76}$-PBEP86-D4	0.17	0.10	0.28	0.20	0.14	0.28	0.23	0.20	0.17	0.14	0.09	0.09	0.05
xMOS$_{78}$-PBEP86-D4	0.19	0.14	0.20	0.30	0.11	0.28	0.23	0.20	0.17	0.16	0.16	0.15	0.07
noDispSD-PBEP86	0.40	0.34	0.37	0.62	0.29	0.56	0.52	0.48	0.44	0.40	0.30	0.18	0.07
xnoDispSD-PBEP86	0.36	0.29	0.36	0.55	0.27	0.53	0.47	0.42	0.38	0.34	0.25	0.17	0.07
revDOD-PBEP86-D4	0.19	0.16	0.19	0.31	0.11	0.32	0.25	0.20	0.17	0.15	0.13	0.13	0.07
xDOD$_{72}$-PBEP86-D4	0.20	0.16	0.19	0.32	0.12	0.34	0.26	0.20	0.17	0.15	0.14	0.13	0.07
Pr2SCAN$_{69}$-D4	0.17	0.16	0.20	0.24	0.12	0.27	0.23	0.20	0.18	0.16	0.11	0.05	0.02

Appendix L: Figures

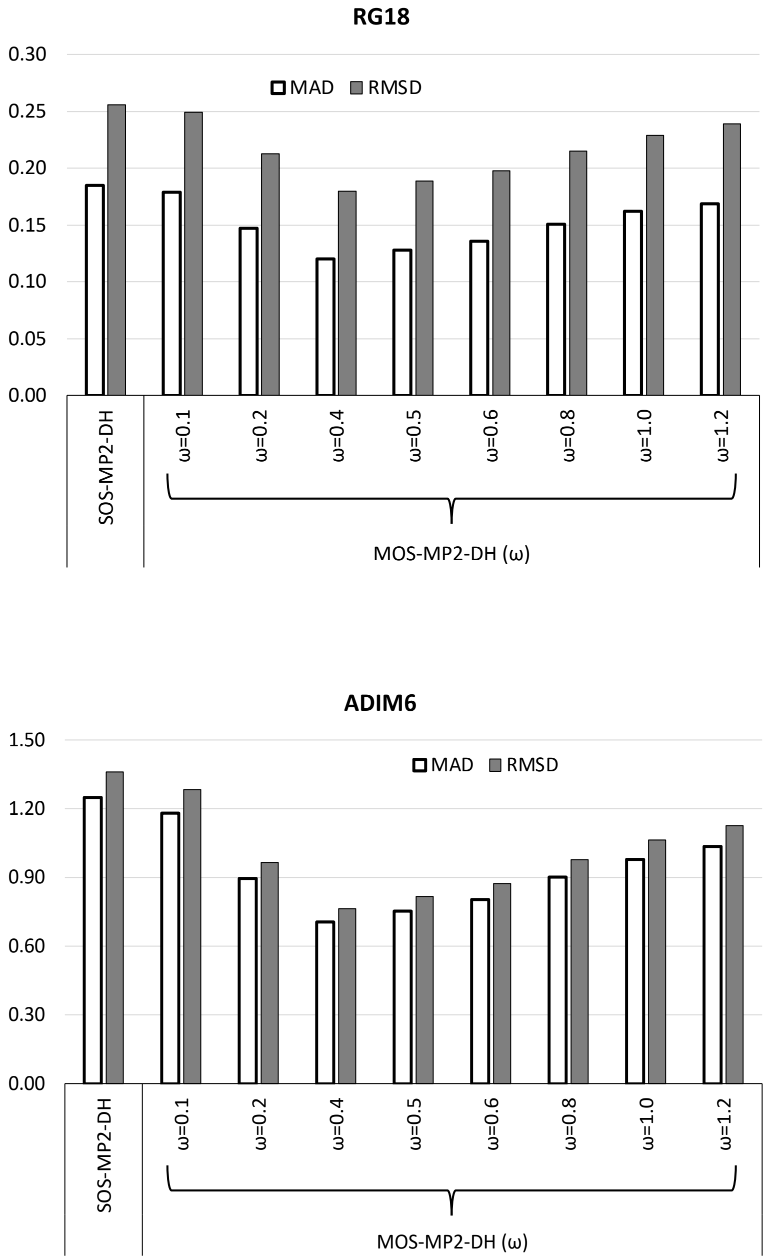

FIG. S1. Effect of using different distance-dependent scaling factors (ω) in MOS_{69}-PBEP86. For comparison, results for the SOS-MP2-based double hybrid, $noDispOD_{69}$-PBEP86, are also presented.

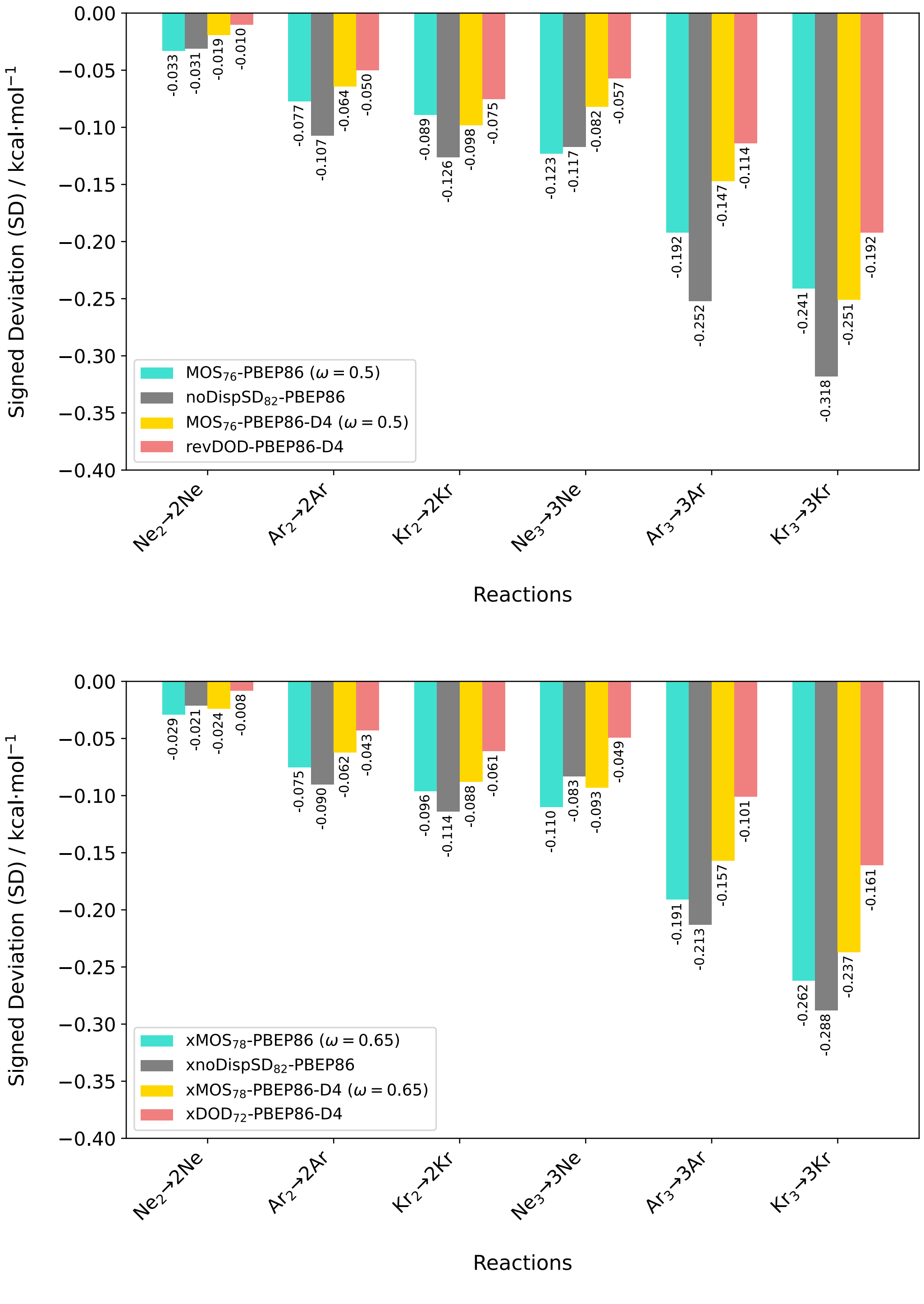

FIG. S2. Signed deviations (SD in kcal·mol^{-1}) of the interaction energies of the Ne, Ar, and Kr dimers and trimers relative to the CCSD(T)/CBS reference data for dispersion-corrected and uncorrected MOS-DHs, gDHs, and xDHs.

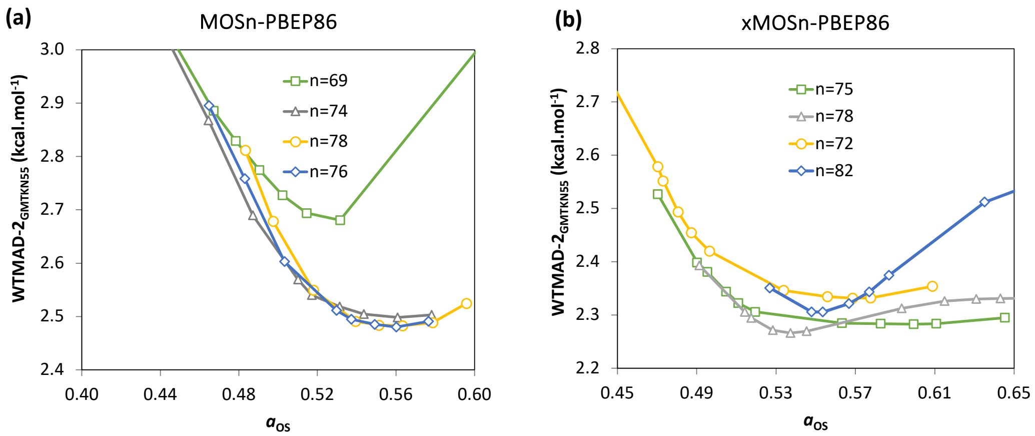

FIG. S3. Dependence of WTMAD-2_{GMTKN55} (kcal·mol^{-1}) on the MOS-PT2 correlation parameter (a_{OS}) of (a) MOS$_n$-PBEP86 and (b) xMOS$_n$-PBEP86. The number "n" in each case represents the percentages of HF exchange used (i.e., $n = 100a_X$).

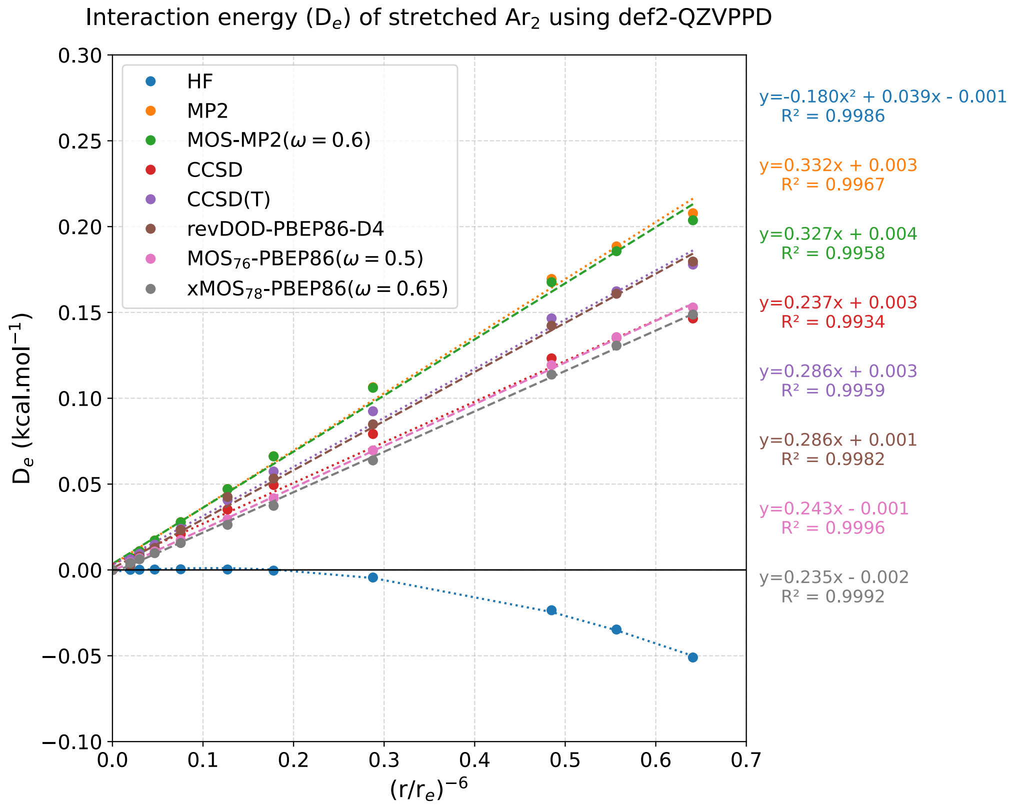

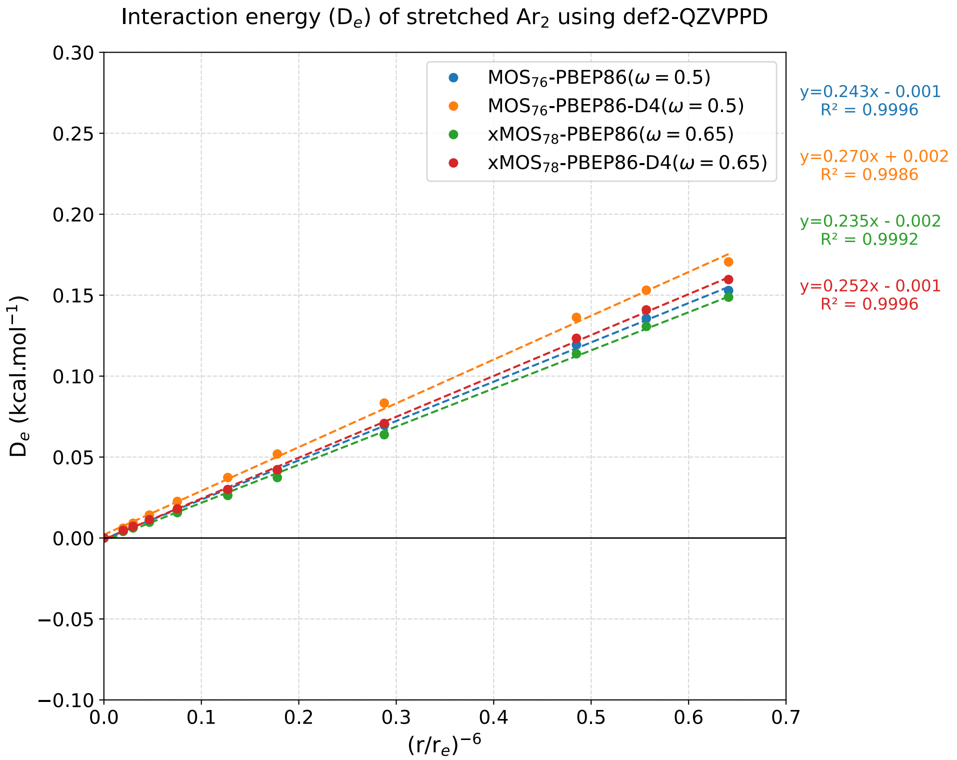

FIG. S4. Interaction energy of stretched Ar_2 plotted against r^{-6} using different methods with the def2-QZVPPD basis set. The fitting equations and corresponding coefficients of determination are listed on the right side of the plot with the matching color.

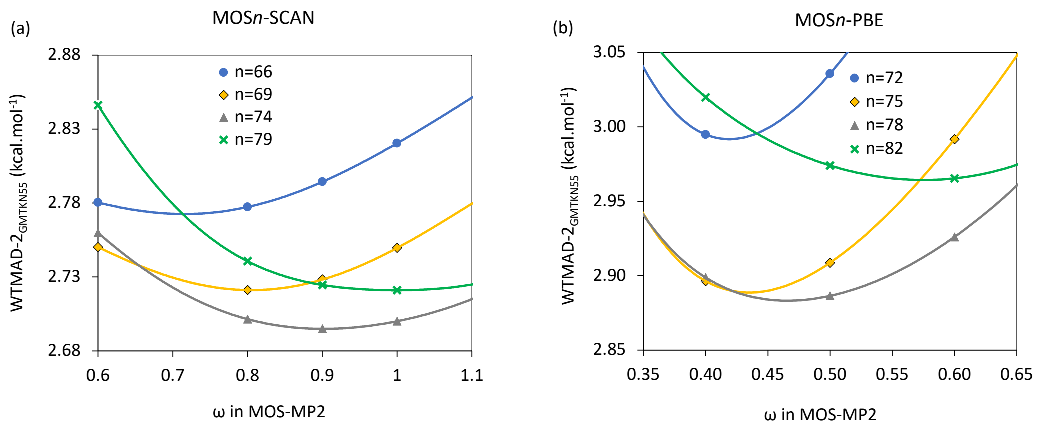

FIG. S5. Dependence of total WTMAD-2_{GMTKN55} (kcal·mol^{-1}) on the MOS-MP2 attenuation parameter (ω) of MOS$_n$-SCAN (a) and MOS$_n$-PBE (b). The number "n" in MOS$_n$-SCAN and MOS$_n$-PBE represent the percentages of HF exchange (i.e., $n = 100a_X$). The number "m" represents the percentage of HF exchange used in the reference orbitals of MOS-XYG@B$_m$LYP.

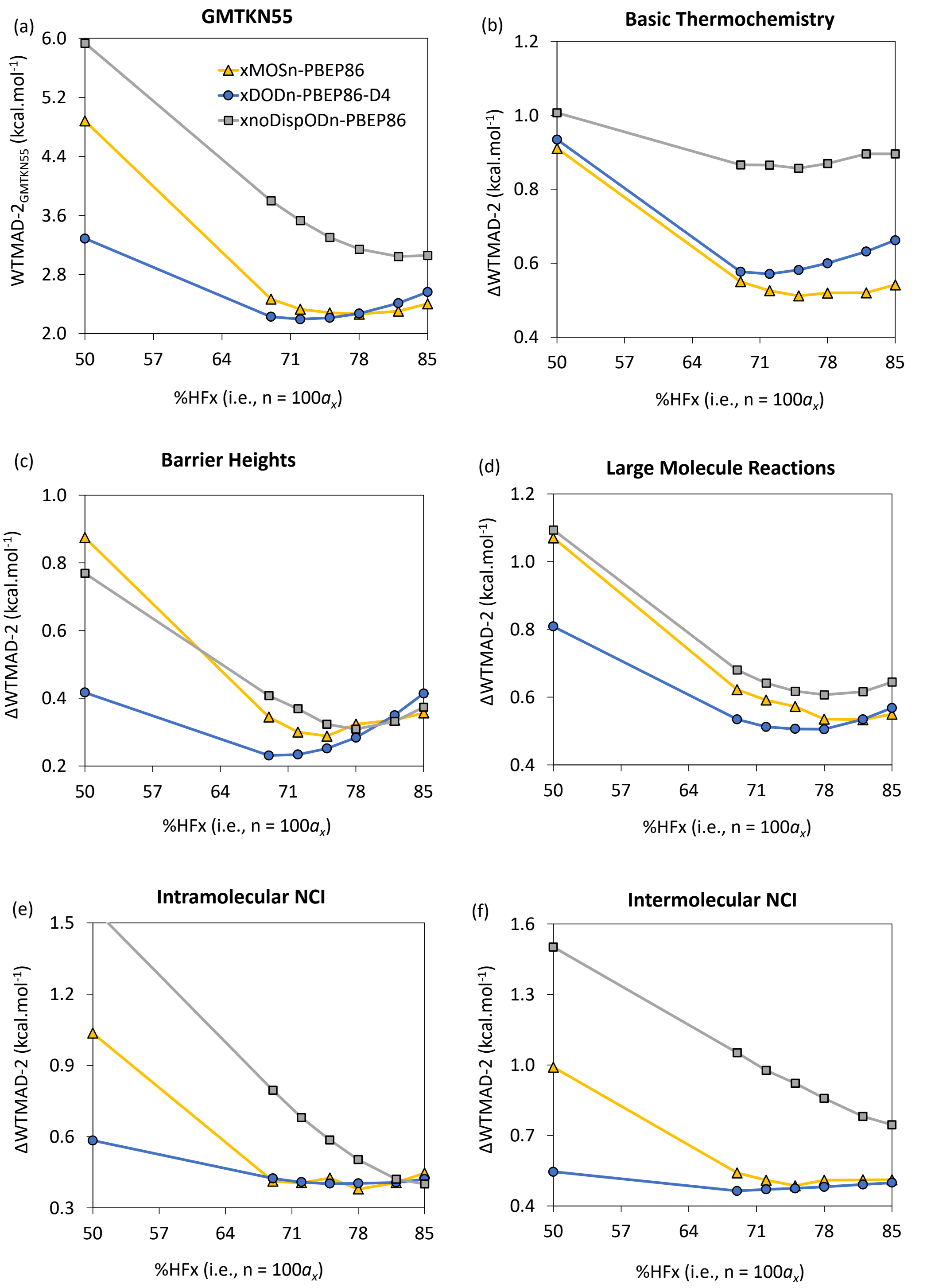

FIG. S6. (a) Variation of total WTMAD-2 (in kcal·mol^{-1}) with the percentage of HF exchange in xMOS$_n$-PBEP86 (yellow), xnoDispSD$_n$-PBEP86 (gray), and xDOD$_n$-PBEP86-D4 (blue) for the full GMTKN55 benchmark. (b-f) Corresponding variations in ΔWTMAD-2 for basic thermochemistry reactions, barrier heights, reactions involving large molecules, intramolecular noncovalent interactions, and intermolecular noncovalent interactions, respectively.

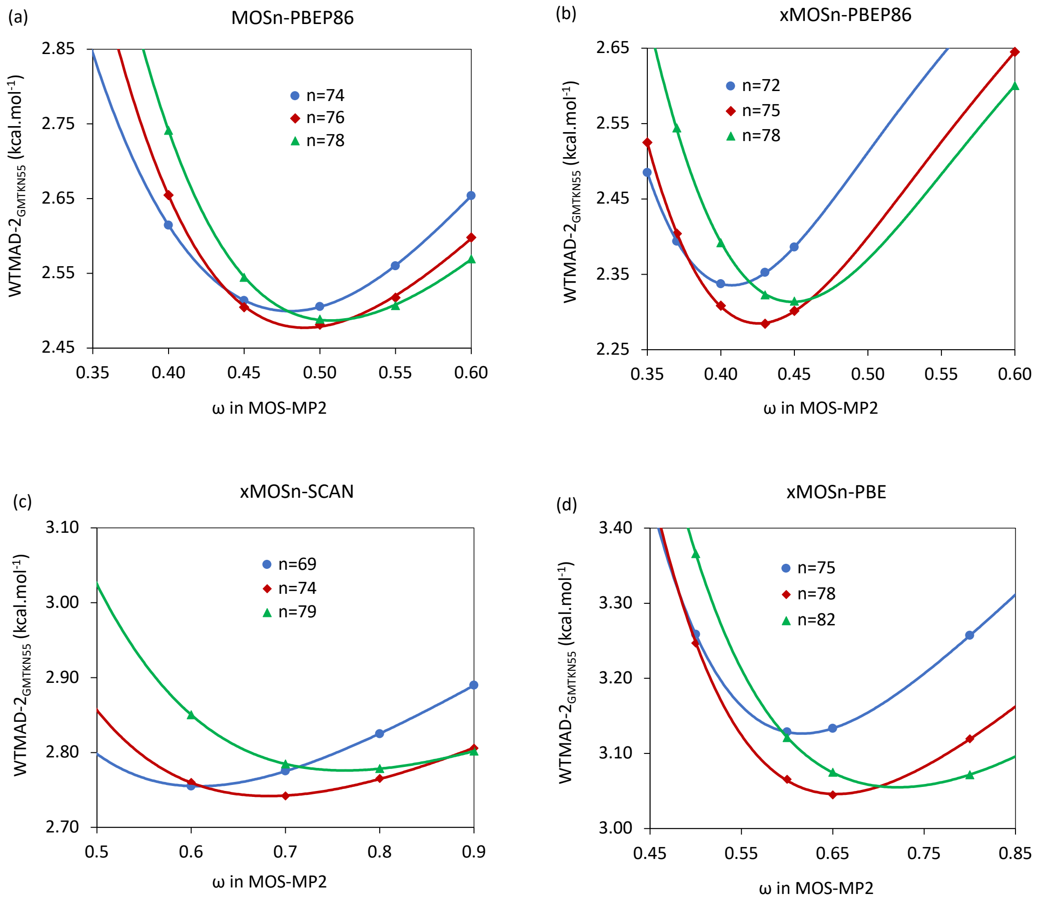

FIG. S7. Dependence of WTMAD-2_{GMTKN55} (kcal·mol^{-1}) on the MOS-MP2 attenuation parameter (ω) of (a) MOS$_n$-PBEP86, (b) xMOS$_n$-PBEP86, (c) MOS$_n$-SCAN, and (d) MOS$_n$-PBE . The number "n" in each case represents the percentages of HF exchange (i.e., $n = 100a_X$). During optimization the a_C + a_{OS} = 1.0 constraint was used.

REFERENCES

[1] L. Goerigk, A. Hansen, C. Bauer, S. Ehrlich, A. Najibi, and S. Grimme, Phys. Chem. Chem. Phys. **19**, 32184 (2017).
[2] H. Yu and D. G. Truhlar, J. Chem. Theory Comput. **11**, 2968 (2015).
[3] Y. Zhao, B. J. Lynch, and D. G. Truhlar, Phys. Chem. Chem. Phys. **7**, 43 (2005).
[4] Y. Zhao, N. González-García, and D. G. Truhlar, J. Phys. Chem. A **109**, 2012 (2005).
[5] L. Goerigk and S. Grimme, J. Chem. Theory Comput. **6**, 107 (2010).
[6] Y. Zhao and D. G. Truhlar, Theoretical Chemistry Accounts **120**, 215 (2008).
[7] S. Grimme, The Journal of Chemical Physics **124**, 034108 (2006).
[8] S. Grimme, C. Mück-Lichtenfeld, E.-U. Würthwein, A. W. Ehlers, T. P. M. Goumans, and K. Lammertsma, J. Phys. Chem. A **110**, 2583 (2006).
[9] M. Piacenza and S. Grimme, Journal of Computational Chemistry **25**, 83 (2004).
[10] H. L. Woodcock, H. F. Schaefer, and P. R. Schreiner, J. Phys. Chem. A **106**, 11923 (2002).
[11] P. R. Schreiner, A. A. Fokin, R. A. Pascal, and A. de Meijere, Organic Letters **8**, 3635 (2006).
[12] C. Lepetit, H. Chermette, M. Gicquel, J.-L. Heully, and R. Chauvin, J. Phys. Chem. A **111**, 136 (2007).
[13] J. S. Lee, J. Phys. Chem. A **109**, 11927 (2005).
[14] A. Karton and J. M. Martin, Molecular Physics **110**, 2477 (2012).
[15] Y. Zhao, O. Tishchenko, J. R. Gour, W. Li, J. J. Lutz, P. Piecuch, and D. G. Truhlar, J. Phys. Chem. A **113**, 5786 (2009).
[16] D. Manna and J. M. L. Martin, J. Phys. Chem. A **120**, 153 (2016).
[17] J. Friedrich and J. Hänchen, J. Chem. Theory Comput. **9**, 5381 (2013).
[18] J. Friedrich, J. Chem. Theory Comput. **11**, 3596 (2015).
[19] L. A. Curtiss, K. Raghavachari, G. W. Trucks, and J. A. Pople, The Journal of Chemical Physics **94**, 7221 (1991).
[20] L. A. Curtiss, K. Raghavachari, P. C. Redfern, and J. A. Pople, The Journal of Chemical Physics **106**, 1063 (1997).
[21] L. Goerigk and S. Grimme, J. Chem. Theory Comput. **7**, 291 (2011).
[22] S. Grimme, H. Kruse, L. Goerigk, and G. Erker, Angewandte Chemie International Edition **49**, 1402 (2010).
[23] A. Karton, S. Daon, and J. M. Martin, Chemical Physics Letters **510**, 165 (2011).
[24] Y. Zhao, H. T. Ng, R. Peverati, and D. G. Truhlar, J. Chem. Theory Comput. **8**, 2824 (2012).
[25] D. H. Ess and K. N. Houk, J. Phys. Chem. A **109**, 9542 (2005).
[26] V. Guner, K. S. Khuong, A. G. Leach, P. S. Lee, M. D. Bartberger, and K. N. Houk, J. Phys. Chem. A **107**, 11445 (2003).
[27] T. C. Dinadayalane, R. Vijaya, A. Smitha, and G. N. Sastry, J. Phys. Chem. A **106**, 1627 (2002).
[28] L. Goerigk and R. Sharma, Canadian Journal of Chemistry **94**, 1133 (2016).
[29] A. Karton, R. J. O'Reilly, B. Chan, and L. Radom, J. Chem. Theory Comput. **8**, 3128 (2012).
[30] A. Karton, R. J. O'Reilly, and L. Radom, J. Phys. Chem. A **116**, 4211 (2012).
[31] S. N. Steinmann, G. Csonka, and C. Corminboeuf, J. Chem. Theory Comput. **5**, 2950 (2009).
[32] H. Krieg and S. Grimme, Molecular Physics **108**, 2655 (2010).
[33] R. Sure, A. Hansen, P. Schwerdtfeger, and S. Grimme, Phys. Chem. Chem. Phys. **19**, 14296 (2017).
[34] L.-J. Yu and A. Karton, Chemical Physics **441**, 166 (2014).
[35] E. R. Johnson, P. Mori-Sánchez, A. J. Cohen, and W. Yang, The Journal of Chemical Physics **129**, 204112 (2008).
[36] S. Grimme, M. Steinmetz, and M. Korth, The Journal of Organic Chemistry **72**, 2118 (2007).
[37] R. Huenerbein, B. Schirmer, J. Moellmann, and S. Grimme, Phys. Chem. Chem. Phys. **12**, 6940 (2010).
[38] F. Neese, T. Schwabe, S. Kossmann, B. Schirmer, and S. Grimme, J. Chem. Theory Comput. **5**, 3060 (2009).
[39] D. Gruzman, A. Karton, and J. M. L. Martin, J. Phys. Chem. A **113**, 11974 (2009).
[40] M. K. Kesharwani, A. Karton, and J. M. L. Martin, J. Chem. Theory Comput. **12**, 444 (2016).
[41] S. Kozuch, S. M. Bachrach, and J. M. Martin, J. Phys. Chem. A **118**, 293 (2014).
[42] S. Grimme, Angewandte Chemie International Edition **45**, 4460 (2006).
[43] T. Schwabe and S. Grimme, Phys. Chem. Chem. Phys. **9**, 3397 (2007).
[44] U. R. Fogueri, S. Kozuch, A. Karton, and J. M. Martin, J. Phys. Chem. A **117**, 2269 (2013).
[45] D. Řeha, H. Valdés, J. Vondrášek, P. Hobza, A. Abu-Riziq, B. Crews, and M. S. de Vries, Chemistry – A European Journal **11**, 6803 (2005).
[46] L. Goerigk, A. Karton, J. M. L. Martin, and L. Radom, Phys. Chem. Chem. Phys. **15**, 7028 (2013).
[47] G. I. Csonka, A. D. French, G. P. Johnson, and C. A. Stortz, J. Chem. Theory Comput. **5**, 679 (2009).
[48] H. Kruse, A. Mladek, K. Gkionis, A. Hansen, S. Grimme, and J. Sponer, J. Chem. Theory Comput. **11**, 4972 (2015).
[49] S. Grimme, J. Antony, S. Ehrlich, and H. Krieg, The Journal of Chemical Physics **132**, 154104 (2010).
[50] K. U. Lao, R. Schäffer, G. Jansen, and J. M. Herbert, J. Chem. Theory Comput. **11**, 2473 (2015).
[51] S. Kozuch and J. M. L. Martin, J. Chem. Theory Comput. **9**, 1918 (2013).
[52] J. Řezáč, K. E. Riley, and P. Hobza, J. Chem. Theory Comput. **8**, 4285 (2012).
[53] D. Setiawan, E. Kraka, and D. Cremer, J. Phys. Chem. A **119**, 1642 (2015).
[54] P. Jurečka, J. Šponer, J. Černý, and P. Hobza, Phys. Chem. Chem. Phys. **8**, 1985 (2006).
[55] J. Řezáč, K. E. Riley, and P. Hobza, J. Chem. Theory Comput. **7**, 2427 (2011).
[56] V. S. Bryantsev, M. S. Diallo, A. C. T. van Duin, and W. A. I. Goddard, J. Chem. Theory Comput. **5**, 1016 (2009).
[57] G. Santra, N. Sylvetsky, and J. M. L. Martin, J. Phys. Chem. A **123**, 5129–5143 (2019).
[58] G. Santra, M. Cho, and J. M. L. Martin, J. Phys. Chem. A **125**, 4614 (2021).
[59] G. Santra, M. Cho, and J. M. L. Martin, J. Phys. Chem. A **128**, 974 (2024).
[60] G. Santra, E. Semidalas, and J. M. L. Martin, The Journal of Physical Chemistry Letters **12**, 9368 (2021).
[61] L. Wittmann, H. Neugebauer, S. Grimme, and M. Bursch, The Journal of Chemical Physics **159**, 224103 (2023).
[62] G. Santra, E. Semidalas, N. Mehta, A. Karton, and J. M. Martin, Phys. Chem. Chem. Phys. **24**, 25555 (2022), arXiv:2208.01500.
